# Comprehensive immunogenomic landscape analysis of prognosis-related genes in head and neck cancer

**DOI:** 10.1038/s41598-020-63148-8

**Published:** 2020-04-14

**Authors:** Lei Li, Xiao-Li Wang, Qian Lei, Chuan-Zheng Sun, Yan Xi, Ran Chen, Yong-Wen He

**Affiliations:** 1 Department of Head and Neck Surgery Section II, the Third Affiliated Hospital of Kunming Medical University, 519 Kunzhou Road, Kunming, China; 2 Radiation Therapy Center, the Third Affiliated Hospital of Kunming Medical University, 519 Kunzhou Road, Kunming, China; 3 Department of Dental Research, The Affiliated Stomatological Hospital of Kunming Medical University, Yunnan, China

**Keywords:** Cancer, Computational biology and bioinformatics, Genetics, Immunology, Biomarkers, Oncology

## Abstract

Head and neck cancer is the sixth most common malignancy around the world, and 90% of cases are squamous cell carcinomas. In this study, we performed a systematic investigation of the immunogenomic landscape to identify prognostic biomarkers for head and neck squamous cell carcinoma (HNSCC). We analyzed the expression profiles of immune‐related genes (IRGs) and clinical characteristics by interrogating RNA-seq data from 527 HNSCC patients in the cancer genome atlas (TCGA) dataset, including 41 HPV+ and 486 HPV− samples. We found that differentially expressed immune genes were closely associated with patient prognosis in HNSCC by comparing the differences in gene expression between cancer and normal samples and performing survival analysis. Gene Ontology (GO) and Kyoto Encyclopedia of Genes and Genomes (KEGG) pathway analyses were performed to annotate the biological functions of the differentially expressed immunogenomic prognosis-related genes. Two additional cohorts from the Oncomine database were used for validation. 65, 56 differentially expressed IRGs was associated with clinical prognosis in total and HPV^-^ samples, respectively. Furthermore, we extracted 10, 11 prognosis-related IRGs from 65, 56 differentially expressed IRGs, respectively. They were significantly correlated with clinical prognosis and used to construct the prognosis prediction models. The multivariable ROC curves (specifically, the AUC) were used to measure the accuracy of the prognostic models. These genes were mainly enriched in several gene ontology (GO) terms related to immunocyte migration and receptor and ligand activity. KEGG pathway analysis revealed enrichment of pathways related to cytokine−cytokine receptor interactions, which are primarily involved in biological processes. In addition, we identified 63 differentially expressed transcription factors (TFs) from 4784 differentially expressed genes, and 16 edges involving 18 nodes were formed in the regulatory network between differentially expressed TFs and the high-risk survival-associated IRGs. B cell and CD4 T cell infiltration levels were significantly negatively correlated with the expression of prognosis-related immune genes regardless of HPV status. In conclusion, this comprehensive analysis identified the prognostic IRGs as potential biomarkers, and the model generated in this study may enable an accurate prediction of survival.

## Introduction

Head and neck cancer is a common malignancy accounting for 5–10% of cancers worldwide. Head and neck squamous cell carcinoma constitutes 90% of head and neck cancers, which arises from the pharynx, the oral cavity and lip, the ear, the larynx, the nasal cavity, the salivary glands and the paranasal sinuses^1–3^. The tobacco, alcohol use and human papillomavirus (HPV) infection are important causes of head and neck squamous cell carcinoma^4^. HNSCC represents a biologically complex disease process and a heterogeneous collection of tumors in which multiple pathways are altered, leading to the development of HNSCC, and the mechanisms leading to this disease are still not clearly understood^5^. The main treatments for HNSCC include surgery, radiotherapy, and chemotherapy, either alone or in combinations. Despite a multimodal approach, the majority of patients with locally advanced HNSCC develop recurrence or distant metastases, such that the 5-year overall survival does not usually exceed 60%^6^. The locoregionally advanced and distantly metastatic HNSCC cases that are unsuitable for surgery or radiotherapy are significantly associated with a poor prognosis, with an expected survival on the order of 6–10 months^7^.

Extracted from TCGA data, protein-coding genes that paint a molecular portrait of the disease can be helpful tools and can be tested as biomarkers. Many studies analyzing the cellular landscape indicated that several genes were differentially expressed between tumor and healthy tissues. However, the differential expression status of the IRGs in HNSCC has not been revealed by comprehensive analysis.

In recent years, it has been well established that the immune system plays a pivotal role in the control of tumor growth, and it has been suggested that potentially invading cancer cells are held in equilibrium via the immune system^8^. Cutting-edge immunotherapy treatments, which are beneficial to recurrent/metastatic (R/M) HNSCC patients, have recently revolutionized the treatment of multiple cancers^9^. Some clinical trials have demonstrated that immune checkpoint therapy is effective for R/M HNSCC and has less toxicity than other therapies^10^. Interestingly, one of the major advantages of immunotherapy over other forms of systemic cancer therapy is that responses can be quite durable—with clinical benefit sometimes measured in years. Long-term follow-up data for survival outcomes following immunotherapy for R/M HNSCC demonstrated that pembrolizumab exhibited durable antitumor activity and a high survival rate in advanced HNSCC patients. The overall response rate was 18%, while 85% of responses lasted 6 months or more, and 71% of responses lasted a year or more^11^. Many studies have emphasized that more immunological biomarkers need to be discovered to provide prognostic information and facilitate clinical decision-making. Therefore, identifying immune-related biomarkers to manipulate the immune response and achieve greater clinical benefit for R/M HNSCC patients is key.

In this study, we combined the expression profiles of immune-related genes (IRGs) with clinical information by comparing the differences in gene expression between normal and tumor tissues and performing Cox regression analysis. Furthermore, bioinformatics analysis was utilized to explore the intrinsic regulatory mechanisms of immune-related genes. Our findings reveal the potential clinical application of IRGs as biomarkers in prognosis prediction, and IRGs may represent therapeutic targets for HNSCC immunotherapy.

## Results

### Identification of differentially expressed IRGs

**Figure 1:**
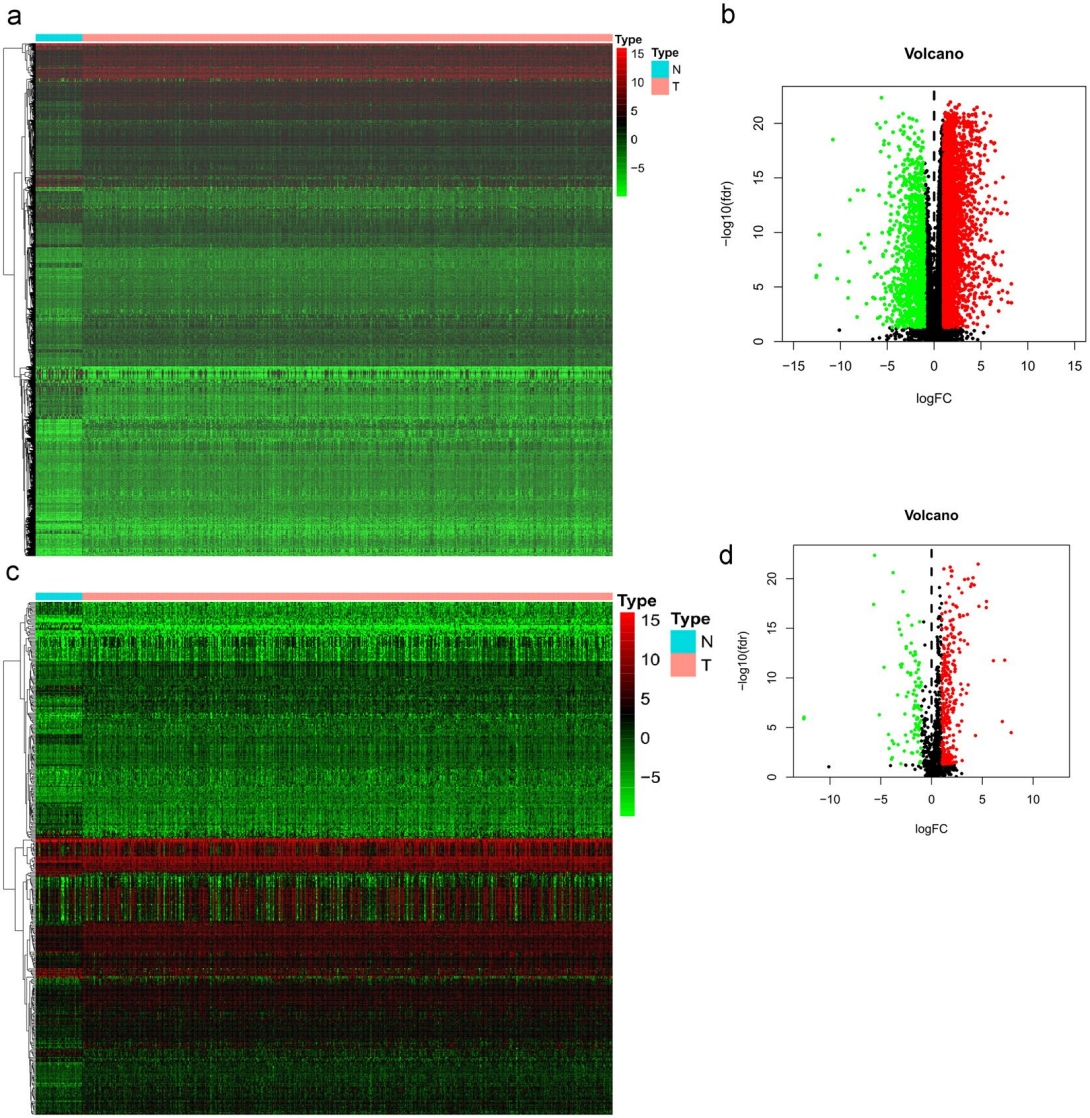
Comparison of gene expression profiles between head and neck squamous cell carcinoma and nontumor tissues. (**a**) Heatmap and (**b**) volcano plot demonstrating differentially expressed genes. (**c**) Heatmap and (**d**) volcano plot demonstrating differentially expressed immune genes. Red represents upregulated differentially expressed genes, blue represents downregulated differentially expressed genes, and black dots (volcano) represent genes that were not differentially expressed.

**Figure 2:**
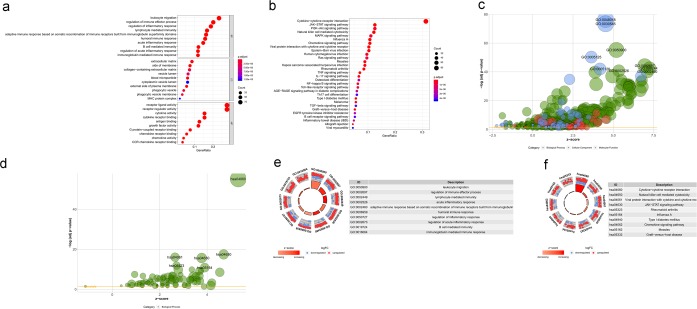
Gene functional enrichment of differentially expressed immune-related genes. Differentially expressed IRGs identified via Gene Ontology (GO) (**a,c)** and KEGG pathway (**b,d**) analyses. In the bubble plot **(c**), green, red and blue bars represent biological processes, cellular components and molecular functions, respectively. The top 10 most significant GO terms (**e**) and Kyoto Encyclopedia of Genes and Genomes pathways (**f**). Nodes in the concentric circle graph represent co-expressed genes clustered in specific biological process terms. The inner sectors with larger size and darker color represent more significant enrichment.

In the present study, we downloaded a total of 56753 genes information from TCGA database including tumor and normal samples. 4784 differentially expressed genes were identified, 3603 of which were upregulated and 1181 of which were downregulated in HNSCC patients (Fig. 1a,b). Furthermore, 399 differentially expressed IRGs were identified from a subset of the 4784 genes, 304 of which were upregulated and 95 of which were downregulated in HNSCC patients (Table 1; Fig. 1c,d). In addition, the functions of the intersecting genes (399 differentially expressed IRGs) were predicted. In the Gene Ontology (GO) and KEGG pathway analyses, 1550 terms and 94 pathways were identified. The top three GO terms were “leukocyte migration (GO:0050900)”, “regulation of immune effector process (GO:0002697)” and “regulation of inflammatory response (GO:0050727)” in terms of biological processes; “extracellular matrix (GO:0030198)”, “side of membrane (GO:0098552)” and “collagen-containing extracellular matrix (GO:0062023)” in terms of cellular components; and “receptor ligand activity (GO:0048018)”, “ receptor regulator activity (GO:0030545)” and “cytokine activity (GO:0005125)” in terms of molecular functions (Fig. 2a,c,e). In the Kyoto Encyclopedia of Genes and Genomes (KEGG) pathways analysis, cytokine-cytokine receptor interaction (hsa04060) was the pathway most often enriched by the differentially expressed IRGs (Fig. 2b,d,f).

**Table 1:** Identification of the differentially expressed immune-related genes (The logFC means log2|fold change | , log F/C > 1 indicated that the expression of genes were up-regulated in HNSCC patients, while log F/C < 1 indicated that expression of genes were down-regulated in HNSCC patients).

Gene	Normal mean	Tumor mean	LogFC	P value	FDR
AZGP1	140.761501	7.469646968	−4.23607	2.14E-05	4.61E-05
FCER1G	11.2589868	26.17050568	1.216865	1.25E-09	5.33E-09
HLA-A	254.911533	613.2837215	1.266558	3.09E-13	2.44E-12
HLA-B	326.464724	877.4184076	1.426338	3.61E-13	2.81E-12
HLA-C	236.984649	555.0795393	1.227901	1.21E-12	8.58E-12
HLA-DOB	0.990856	2.369508193	1.25784	1.27E-08	4.54E-08
HLA-F	10.370698	30.66749407	1.564197	1.31E-12	9.22E-12
HLA-G	1.39598199	6.074506878	2.121487	3.54E-10	1.64E-09
HLA-H	22.4554075	49.17635987	1.130902	1.44E-09	6.03E-09
HSPA2	9.76782464	19.61169521	1.005605	0.000272	0.000498
ICAM1	11.2802693	25.07587424	1.152498	1.17E-08	4.23E-08
IFNG	0.1438456	0.654326879	2.18549	4.87E-08	1.58E-07
KIR2DL4	0.1522916	0.529638836	1.798173	5.58E-06	1.32E-05
KLRC1	0.11234459	0.279755016	1.316233	0.001152	0.001918
LTA	0.21982314	0.521766513	1.247061	1.24E-06	3.23E-06
MICB	0.89624206	3.786667263	2.078968	4.86E-17	9.00E-16
PSMD2	39.4400045	91.67299729	1.216837	5.96E-25	9.64E-22
RELB	7.44585989	15.18863955	1.028482	2.08E-11	1.18E-10
TAP1	24.7771851	74.2062648	1.582529	2.56E-14	2.51E-13
TAP2	5.75960252	13.95711697	1.27696	8.25E-16	1.12E-14
IFI30	0.18085808	0.521648619	1.52822	5.21E-17	9.54E-16
PROCR	6.49854461	24.59981521	1.920459	2.15E-16	3.40E-15
ULBP3	1.29958122	2.880656553	1.148351	2.54E-08	8.61E-08
ULBP2	3.1334658	12.91807779	2.04356	5.31E-18	1.31E-16
ULBP1	0.13572911	1.001890626	2.883923	7.45E-11	3.83E-10
RAET1E	13.2144682	3.93391552	−1.74808	3.46E-08	1.15E-07
PDIA2	0.05969777	1.199043433	4.328063	2.80E-05	5.92E-05
CXCL14	122.268502	372.1008438	1.605641	0.000748	0.00128
SLPI	2610.15495	830.9769458	−1.65126	1.48E-08	5.24E-08
CXCL10	15.196852	124.3922942	3.033053	2.83E-10	1.33E-09
CXCL9	9.24006951	49.69692373	2.427181	4.01E-09	1.57E-08
CXCL11	2.02329743	24.56421975	3.601778	9.19E-11	4.66E-10
CXCL12	15.0119696	5.452086988	−1.46123	4.96E-07	1.38E-06
CXCL13	2.64456939	19.00938842	2.845607	1.00E-15	1.34E-14
CXCL2	9.72385442	4.565688464	−1.0907	0.017695	0.024321
XCL1	0.28104112	1.248440254	2.151274	5.61E-13	4.22E-12
DEFB1	176.677144	53.88491009	−1.71316	3.51E-10	1.63E-09
TMSB10	1092.64225	2821.300725	1.368539	6.92E-19	2.19E-17
LCN2	447.352172	159.2711221	−1.48993	2.39E-08	8.15E-08
S100A9	15432.446	6667.03957	−1.21085	3.22E-06	7.86E-06
S100A8	6148.25986	2377.261991	−1.37088	2.42E-06	6.04E-06
HTN3	1113.90311	0.186136307	−12.547	2.91E-07	8.39E-07
MMP12	2.4760227	38.28779427	3.950788	2.99E-22	3.14E-20
PTGDS	20.3392441	7.284519543	−1.48136	1.26E-09	5.36E-09
TMSB15A	0.31540397	2.161507024	2.776765	1.03E-05	2.34E-05
DEFB126	0.05038056	0.226744114	2.170126	0.020469	0.027794
S100A5	0.22413643	0.486194597	1.117157	0.008856	0.012838
S100A1	28.6642829	2.833404063	−3.33865	1.13E-09	4.85E-09
HTN1	234.309738	0.038339574	−12.5773	4.20E-07	1.18E-06
S100A14	1032.71172	454.45533	−1.18423	1.25E-06	3.25E-06
TINAGL1	7.20176813	19.21614587	1.415896	4.63E-10	2.11E-09
WFDC2	427.5378	29.38590271	−3.86286	0.006801	0.010059
TGFB1	12.6913796	45.44074526	1.840138	3.54E-25	6.30E-22
MMP9	4.68250673	79.57169061	4.086902	3.60E-23	7.44E-21
APOBEC3G	1.42821266	3.614852766	1.339726	2.69E-07	7.79E-07
FABP6	1.10315656	3.75998828	1.769091	2.90E-05	6.12E-05
RBP1	7.59289009	37.30236814	2.296546	5.35E-16	7.61E-15
PLAU	17.1784923	110.1589636	2.680912	1.02E-21	8.42E-20
IL1B	3.71074115	13.10607739	1.820457	1.08E-06	2.85E-06
PAEP	0.03142237	1.32057542	5.393231	2.05E-19	7.58E-18
MX1	11.8010874	33.38941718	1.500471	2.19E-08	7.53E-08
DDX58	4.17161375	14.29679051	1.777014	5.30E-14	4.91E-13
SFTPA2	0.76667193	0.336438314	−1.18827	1.59E-10	7.76E-10
LBP	1.64869075	0.736595416	−1.16238	0.024851	0.033252
RBP4	2.27829793	0.912345169	−1.3203	1.56E-08	5.49E-08
NOX4	0.21387194	0.915830479	2.098333	7.43E-17	1.30E-15
LTF	917.674427	36.01701431	−4.67123	1.10E-12	7.87E-12
FABP7	5.56457715	0.157924359	−5.13897	1.63E-07	4.88E-07
FABP3	44.1770711	6.908420351	−2.67687	0.00131	0.002165
OASL	2.62095302	17.0465449	2.701316	2.19E-16	3.45E-15
DUOX1	28.8369572	14.15939772	−1.02616	4.70E-08	1.53E-07
FABP12	0.52989973	0.256180969	−1.04856	0.000629	0.001092
PI15	0.49099368	1.362655272	1.472644	3.64E-09	1.43E-08
APOD	46.1811346	8.244769222	−2.48575	5.73E-15	6.43E-14
ORM2	0.0788061	0.225477574	1.516605	4.84E-05	9.88E-05
CTSG	4.33606479	1.330628377	−1.70428	2.12E-05	4.57E-05
PML	7.27390668	15.30806882	1.07349	5.06E-13	3.84E-12
CYBB	4.16431981	8.686060183	1.060621	0.000129	0.000248
ISG20	2.49483593	5.24894123	1.073082	1.48E-06	3.83E-06
TFRC	11.9428901	37.92448056	1.666977	3.11E-13	2.46E-12
IFIH1	5.25357518	15.36513689	1.548289	8.48E-12	5.16E-11
IDO1	3.55528778	16.98751225	2.256436	5.29E-09	2.02E-08
ADIPOQ	4.43687949	0.090920784	−5.60879	2.32E-27	4.12E-23
STAT1	29.7280212	89.78982168	1.594728	6.47E-14	5.87E-13
TNFSF10	37.0843358	85.95761174	1.212815	4.04E-08	1.32E-07
CCL20	6.80143542	22.5198056	1.727283	1.20E-06	3.13E-06
SOCS1	2.54808466	11.06232065	2.118169	8.88E-20	3.59E-18
IL15	0.39916272	0.874389501	1.131299	5.20E-06	1.23E-05
CHIT1	0.20032363	2.170568122	3.437668	7.40E-10	3.26E-09
VEGFA	4.16606964	10.34122599	1.311648	2.61E-12	1.75E-11
ISG15	21.9969809	318.5958413	3.85635	6.27E-22	5.66E-20
DHX58	2.5898532	5.375063939	1.053412	6.25E-11	3.25E-10
TNFAIP3	10.9562653	24.03152342	1.133172	2.79E-09	1.12E-08
TFR2	0.17390547	0.546720142	1.652499	4.38E-06	1.05E-05
MUC4	10.5167619	3.130648606	−1.74816	5.09E-05	0.000104
F2R	4.76270265	12.43949049	1.385075	4.74E-12	3.03E-11
MAPT	2.22286807	0.283387734	−2.97157	2.79E-16	4.27E-15
LYZ	1480.74523	112.8877688	−3.71336	0.00021	0.000391
CCL5	11.7542346	49.97424577	2.088004	7.64E-10	3.36E-09
ITGAV	11.7548612	28.92172488	1.298896	5.51E-14	5.08E-13
TLR8	0.30481515	0.7045093	1.208684	0.000726	0.001247
GNLY	1.87734623	7.933403211	2.079245	4.88E-13	3.71E-12
EIF2AK2	3.79883745	10.20996947	1.426349	5.71E-20	2.46E-18
BST2	37.5941515	254.1483336	2.757091	1.38E-18	4.07E-17
PLA2G2A	38.820626	7.929581253	−2.29151	1.63E-18	4.67E-17
ADAR	23.6630778	47.86163445	1.016232	4.46E-18	1.14E-16
MX2	1.7639436	5.455379419	1.628875	9.28E-10	4.03E-09
MSR1	0.87519781	1.910406496	1.126199	5.59E-08	1.80E-07
SLC11A1	0.34450751	1.21576439	1.819256	5.33E-19	1.75E-17
DMBT1	65.6409166	3.617668986	−4.18146	0.000676	0.001168
DES	728.159849	123.9189978	−2.55486	2.51E-05	5.35E-05
TNFRSF10B	7.03680752	15.75748764	1.163045	4.48E-15	5.19E-14
APOBEC3H	0.24598789	0.706353938	1.521804	4.06E-07	1.14E-06
SPINK5	354.298193	44.99588064	−2.9771	1.07E-12	7.66E-12
TNFSF11	0.10591856	0.530948712	2.325617	1.65E-12	1.14E-11
CLDN4	114.276557	40.59751217	−1.49307	1.23E-10	6.10E-10
CCL28	24.4864213	1.584078293	−3.95027	0.010034	0.014398
IRF7	10.2207731	24.55095099	1.264275	6.17E-13	4.60E-12
IL7R	2.27764192	8.021914076	1.816406	1.44E-11	8.46E-11
IL1A	4.34170044	22.08057361	2.346446	9.64E-09	3.52E-08
PTX3	7.05171955	1.821522249	−1.95283	0.00205	0.003284
PTGS2	2.97256793	10.74320113	1.853642	0.000581	0.001014
PROC	0.18503733	1.331676726	2.847356	1.62E-16	2.66E-15
NDRG1	91.6022532	241.7426842	1.400017	3.15E-11	1.72E-10
IRF9	0.48277151	1.48917545	1.625101	3.63E-16	5.40E-15
ABCC4	1.19897173	2.407151667	1.005529	3.41E-10	1.58E-09
PLSCR1	12.0066205	24.15015248	1.008202	1.79E-10	8.71E-10
RSAD2	1.99533893	12.91988771	2.694888	1.03E-14	1.09E-13
PDGFRB	6.27611021	17.8452768	1.5076	6.19E-12	3.87E-11
PDCD1	0.7684706	1.57420676	1.034563	0.021445	0.029014
AQP9	0.59654601	1.638747522	1.457888	2.73E-11	1.51E-10
FASLG	0.32324901	0.724487013	1.164314	0.024377	0.03267
BIRC5	5.05117624	20.54924905	2.024394	2.61E-24	1.53E-21
OAS1	11.2092353	23.35089274	1.05879	7.05E-07	1.91E-06
TNFSF4	0.17675193	1.549628874	3.132125	1.75E-18	4.99E-17
NOS1	2.23283169	0.689385368	−1.69549	2.59E-08	8.76E-08
ACTA1	1981.05982	135.3248897	−3.87177	0.000104	0.000202
CCL26	1.01014639	4.561715584	2.175012	0.000101	0.000198
CCR8	0.17349441	0.734053595	2.080996	2.32E-10	1.11E-09
CCL2	34.9686388	11.61449106	−1.59014	5.29E-09	2.02E-08
CCL7	0.18927901	0.657953341	1.797471	1.96E-09	8.08E-09
CCL3	2.14816002	4.902955744	1.19055	7.68E-08	2.42E-07
CCL11	0.55157223	2.904377118	2.396607	1.38E-19	5.33E-18
CCL23	1.12326388	0.273743401	−2.0368	6.30E-12	3.93E-11
CXCR4	9.22252573	19.16025406	1.054883	0.000386	0.000692
LTBP1	6.62487458	25.99529833	1.972286	1.68E-14	1.71E-13
PPARG	2.88842354	0.756024662	−1.93378	2.16E-17	4.49E-16
MIF	26.9699739	57.37594282	1.089092	1.10E-13	9.54E-13
CD86	1.67553761	3.655170224	1.125315	6.99E-10	3.09E-09
OLR1	0.72531218	3.224972232	2.152613	3.30E-10	1.54E-09
RNASE2	0.34609099	0.725574867	1.067973	8.35E-07	2.24E-06
CD79A	3.16021095	10.3146119	1.706597	0.023568	0.031671
BLNK	10.4408627	4.286057645	−1.28452	1.20E-10	5.99E-10
VAV2	3.81897612	14.93115698	1.967068	2.07E-23	5.32E-21
RAC2	8.0638533	28.71382317	1.832204	7.02E-18	1.67E-16
RAC3	3.49005231	11.12945643	1.673063	2.03E-08	7.03E-08
FOS	338.296862	136.589998	−1.30844	1.96E-12	1.34E-11
CARD11	1.25857938	3.46194499	1.459787	2.19E-06	5.50E-06
CD19	0.23053819	0.740556011	1.683603	0.001518	0.002482
PIK3R1	8.78076848	4.252691387	−1.04597	6.98E-07	1.89E-06
PIK3CD	2.3298192	6.209533165	1.414267	4.14E-17	7.84E-16
AKT3	1.09039538	2.369801878	1.119915	6.20E-07	1.69E-06
CD22	0.25129293	0.539412325	1.102018	0.012393	0.017507
CD72	0.35093164	0.830922486	1.243524	3.41E-08	1.13E-07
IFITM1	60.1765068	172.7061256	1.521047	2.55E-10	1.21E-09
IGHE	0.2414842	0.771029039	1.674856	0.001735	0.002811
IGHG1	86.8715439	698.6877831	3.007692	1.24E-08	4.44E-08
IGHG2	144.085491	642.3323018	2.156395	2.53E-05	5.39E-05
IGHG3	30.1498131	193.1365392	2.6794	2.20E-06	5.52E-06
IGHG4	71.4587322	410.6246363	2.522638	2.13E-06	5.38E-06
IGHM	25.724065	113.9007551	2.146587	0.000581	0.001014
IGHV1-18	28.0447833	116.6576854	2.056477	0.006306	0.009362
IGHV1-24	9.20112685	50.8718685	2.466986	0.000875	0.001484
IGHV1-69	4.30104966	21.78746724	2.340738	0.005635	0.008425
IGHV2-26	5.85552155	20.18384448	1.785331	0.03092	0.04069
IGHV2-5	2.25448061	8.638621574	1.938006	0.010052	0.014422
IGHV2-70	4.16961628	17.08320458	2.034592	0.007378	0.010847
IGHV3-11	13.5472501	48.65714917	1.844652	0.012999	0.018306
IGHV3-15	25.8863216	62.98550917	1.28283	0.033187	0.043404
IGHV3-20	0.91442044	4.904326758	2.423126	0.020563	0.027907
IGHV3-21	15.7078204	56.99584537	1.859374	0.003813	0.005856
IGHV3-23	41.3186634	126.6624015	1.616123	0.007178	0.010576
IGHV3-30	20.7333208	62.45990567	1.590979	0.031063	0.04086
IGHV3-33	12.4224505	33.34431749	1.424491	0.021303	0.028845
IGHV3-48	7.11143582	14.96467857	1.073349	0.009273	0.013399
IGHV3-53	3.91411303	11.4157559	1.544269	0.024646	0.033
IGHV3-64	0.8308217	6.716016837	3.014995	0.013989	0.019583
IGHV3-7	1.23334926	2.669893683	1.114201	0.035765	0.046491
IGHV4-34	10.5658183	42.39739749	2.004571	0.004522	0.00686
IGHV4-39	30.0219714	104.3604529	1.797484	0.013882	0.019448
IGHV4-59	15.9127994	60.28010631	1.921494	0.028581	0.037839
IGHV5-51	42.2463818	136.4318714	1.691281	0.020611	0.027966
IGHV6-1	1.13432516	2.954858673	1.381255	0.012314	0.017404
IGKC	310.677637	922.6267517	1.570329	0.00422	0.006436
IGKJ5	1.28525265	4.612672177	1.843551	0.003279	0.005084
IGKV1-12	0.99550699	2.573234524	1.37008	0.009952	0.014292
IGKV1-16	11.9177292	41.34892335	1.79474	0.018027	0.024752
IGKV1-5	61.956309	179.5210393	1.53483	0.017269	0.023778
IGKV2D-29	3.9638841	13.4372983	1.761256	0.018362	0.025169
IGKV3-11	49.2704227	151.7922943	1.623305	0.003912	0.005994
IGKV3-15	25.5356016	77.71487129	1.605681	0.017013	0.023458
IGKV3-20	85.2825162	278.3799343	1.706733	0.004424	0.006724
IGKV4-1	68.2739321	202.0626519	1.565396	0.0065	0.009636
IGLC2	151.274685	406.896779	1.427492	0.003367	0.005211
IGLC3	104.742295	275.0896662	1.393058	0.005651	0.008449
IGLJ2	0.30590016	0.916362063	1.582857	0.002942	0.004593
IGLV1-40	47.7857769	132.2710552	1.468844	0.01631	0.022585
IGLV1-44	26.0153565	81.27468738	1.643443	0.014452	0.020177
IGLV1-47	25.4370506	66.27028303	1.381431	0.019104	0.026086
IGLV1-50	0.44212857	1.185104284	1.422476	0.033796	0.044112
IGLV1-51	36.3794548	117.9062168	1.696444	0.019806	0.026966
IGLV2-23	34.5489759	97.452528	1.496057	0.028726	0.038017
IGLV3-1	15.9420869	61.57060709	1.949401	0.007619	0.011173
IGLV3-10	11.2153179	46.57915361	2.054214	0.029644	0.039118
IGLV3-19	31.8797257	119.9531001	1.911759	0.009225	0.013333
IGLV3-21	45.4931231	177.2342444	1.961937	0.005405	0.0081
IGLV3-25	48.9199575	111.6293824	1.190222	0.022367	0.030162
IGLV4-69	11.5982515	49.31871381	2.088228	0.031139	0.040953
CMA1	1.98769994	0.403261295	−2.30131	5.89E-10	2.64E-09
CXCL17	117.062679	28.39879541	−2.04338	4.71E-12	3.01E-11
EDN3	6.75141587	0.130648725	−5.69143	9.14E-20	3.66E-18
SAA2	36.8129049	4.564767621	−3.0116	0.031376	0.041211
SEMA3C	7.07077779	15.95431922	1.174006	8.65E-07	2.31E-06
SEMA3G	2.70000023	1.064567734	−1.34269	3.80E-07	1.07E-06
SEMA4F	0.66486563	2.27662203	1.77576	1.15E-20	6.26E-19
SEMA5B	0.08279814	0.385262347	2.218171	2.10E-13	1.71E-12
SEMA6D	0.40114622	0.951156363	1.245554	0.009777	0.01406
SEMA7A	1.76833125	4.814156071	1.444894	6.52E-14	5.91E-13
TNC	23.5188264	115.8420979	2.300272	3.24E-12	2.13E-11
TYMP	45.4861884	141.5798149	1.638115	1.01E-14	1.06E-13
CCRL2	0.34955428	0.735407853	1.073028	2.81E-08	9.44E-08
CX3CR1	1.37980915	0.457133277	−1.59378	4.56E-11	2.42E-10
CXCR3	1.03249804	2.172631282	1.073304	0.018825	0.025735
CYSLTR1	0.81979704	0.336677522	−1.2839	1.74E-11	1.00E-10
CYSLTR2	0.09923963	0.326614036	1.718598	6.95E-11	3.59E-10
EDNRA	2.36183216	5.059874066	1.099195	1.45E-06	3.74E-06
EDNRB	1.9785425	0.952446752	−1.05473	0.003007	0.004688
FPR2	0.1349368	0.443136924	1.715469	3.12E-07	8.93E-07
PLAUR	6.59774454	16.01458766	1.279342	2.78E-13	2.22E-12
PLXNA1	6.91315204	21.17578982	1.615	2.84E-22	3.03E-20
PLXNA3	1.55276495	3.273184314	1.075855	2.85E-12	1.89E-11
PLXNB3	1.02087802	2.067987833	1.018417	1.02E-08	3.72E-08
PLXND1	4.05003807	8.33955808	1.042035	9.74E-12	5.87E-11
ROBO2	0.46743827	0.230123469	−1.02237	4.91E-11	2.59E-10
ADM	23.1966792	46.9101623	1.015982	8.56E-07	2.29E-06
AGT	5.78163796	2.070608106	−1.48142	3.33E-06	8.11E-06
AMH	0.07988846	0.62480892	2.967356	7.61E-07	2.05E-06
ANGPTL7	1.68002292	0.201015181	−3.0631	5.83E-13	4.37E-12
APLN	0.86235123	3.900377306	2.177266	4.54E-16	6.58E-15
ARTN	0.43390071	5.247002259	3.596057	5.63E-23	9.83E-21
BMP1	3.33853928	12.80559466	1.939485	2.61E-24	1.53E-21
BMP2	3.92212365	9.153133226	1.222631	1.08E-07	3.33E-07
BMP3	1.72390668	0.339824676	−2.34282	3.91E-12	2.54E-11
BMP8A	0.06636327	0.526073118	2.986807	3.79E-21	2.45E-19
BMP8B	0.5313324	1.349579758	1.344824	9.28E-12	5.61E-11
BTC	1.78168079	0.579803476	−1.6196	2.21E-14	2.19E-13
CD70	0.30839157	2.87821674	3.22234	1.26E-15	1.65E-14
CGB5	0.00244445	0.360701481	7.205152	1.82E-13	1.50E-12
CGB7	0.0621182	0.274841474	2.145512	4.00E-17	7.62E-16
CGB8	0.00479987	0.327709573	6.093277	2.09E-13	1.70E-12
CLEC11A	2.71065169	9.312385266	1.780511	2.82E-15	3.43E-14
CMTM1	0.19283448	0.524554219	1.443729	4.22E-12	2.72E-11
CMTM3	4.81675736	10.42202404	1.113501	6.69E-12	4.15E-11
CSF2	0.17931906	4.564203615	4.669762	1.47E-19	5.65E-18
CSPG5	0.10920882	0.314350353	1.525284	0.000161	0.000305
DKK1	2.00139197	8.598564529	2.103092	8.49E-08	2.65E-07
EGF	1.40614405	0.353739232	−1.99099	1.08E-05	2.43E-05
EPO	0.11762463	0.588822801	2.323643	1.83E-12	1.25E-11
ESM1	0.88518558	2.453007684	1.4705	5.86E-17	1.06E-15
FAM3B	16.2966982	3.222150502	−2.33848	1.60E-18	4.62E-17
FAM3D	96.035144	6.988279824	−3.78055	5.91E-24	2.29E-21
FGF18	0.7907508	0.26729618	−1.56478	1.54E-06	3.96E-06
FGF19	0.00665932	1.529811499	7.843763	1.35E-05	3.00E-05
FGF7	1.90308134	0.546490517	−1.80007	9.11E-10	3.96E-09
GAST	0.12363756	5.276926614	5.415509	4.03E-20	1.82E-18
GDF10	1.98146718	0.265373097	−2.90048	4.92E-13	3.74E-12
GDF6	0.03060174	0.223556987	2.868957	1.08E-09	4.63E-09
GNRH1	0.18077715	0.466431407	1.367453	1.45E-07	4.38E-07
GREM1	1.0455893	4.727065721	2.176629	5.57E-16	7.89E-15
GREM2	1.58860774	0.179265326	−3.14759	6.58E-17	1.17E-15
GRP	0.41557375	1.600923684	1.945728	1.75E-09	7.28E-09
IFNE	0.15189015	0.691375503	2.186441	2.92E-08	9.79E-08
IFNK	0.19121068	0.647341699	1.759364	1.60E-08	5.62E-08
IL11	0.13642248	3.280347335	4.587695	1.24E-25	3.15E-22
IL12A	0.5559761	0.223027363	−1.3178	0.000136	0.00026
IL17C	0.09787588	0.213580834	1.125757	0.016171	0.022405
IL17D	1.32095958	0.238188898	−2.47141	1.67E-09	6.95E-09
IL1F10	0.32539757	0.783656027	1.268017	0.002822	0.00442
IL1RN	308.866731	92.42516434	−1.74063	2.00E-07	5.90E-07
IL24	0.83979892	9.87783144	3.556079	2.98E-15	3.61E-14
IL33	13.98023	4.709156551	−1.56985	4.15E-14	3.92E-13
IL34	6.63270625	2.842884765	−1.22224	1.73E-11	9.96E-11
INHA	0.07616644	0.214585107	1.494323	1.13E-06	2.98E-06
INHBA	1.02075508	18.84559329	4.206519	4.04E-22	4.04E-20
INHBE	0.11804892	0.278753559	1.239605	4.59E-05	9.41E-05
JAG1	18.6109461	44.3967847	1.254304	4.03E-14	3.81E-13
JAG2	7.3803013	17.14258704	1.215833	7.38E-15	8.07E-14
LHB	0.08880278	0.6175139	2.797795	2.43E-18	6.56E-17
LTBP2	8.59170092	17.58345766	1.033203	8.72E-08	2.72E-07
MDK	26.910812	60.19590711	1.161479	3.14E-06	7.68E-06
MIA	1.98022809	0.548150514	−1.85302	0.002231	0.003555
NGF	0.89179839	2.087460938	1.22696	1.10E-05	2.48E-05
NMB	4.46159827	13.90247985	1.63971	1.82E-12	1.24E-11
NRG1	0.9112157	4.468160248	2.293816	7.30E-13	5.39E-12
NTF3	0.90704007	0.390411652	−1.21617	3.42E-13	2.68E-12
OGN	3.59963628	0.867043828	−2.05367	7.47E-12	4.60E-11
OSM	0.91005352	2.769073982	1.60538	8.63E-08	2.70E-07
PDGFA	2.97864939	7.432678127	1.319224	9.82E-14	8.64E-13
PDGFB	3.10490671	6.567244174	1.080738	1.28E-09	5.42E-09
PDGFD	2.00947478	0.801335171	−1.32634	2.44E-05	5.22E-05
PGF	2.29505443	8.732569631	1.927878	2.04E-17	4.26E-16
PTHLH	5.48040688	63.6062284	3.536813	1.25E-18	3.74E-17
PTN	26.0423208	12.62390556	−1.0447	1.06E-10	5.29E-10
SCG2	0.56913811	1.344286679	1.23999	0.009749	0.014022
SCGB3A1	269.255231	27.49958988	−3.29149	0.000269	0.000494
SLURP1	405.893276	66.07402152	−2.61895	1.32E-07	4.00E-07
SPP1	15.3697514	106.1032656	2.787303	2.42E-14	2.39E-13
STC1	3.23967231	7.683465944	1.245909	6.80E-07	1.85E-06
STC2	0.51361732	4.88917222	3.250824	7.15E-23	1.14E-20
TGFB3	3.54746977	8.099078106	1.190967	9.70E-09	3.54E-08
TNFSF13B	1.24240408	2.870095712	1.207964	5.96E-06	1.40E-05
TNFSF18	0.44962232	1.398115287	1.636698	0.013091	0.01842
TNFSF9	2.76838158	5.596849403	1.015572	2.57E-07	7.46E-07
UCN	0.21565604	0.742440911	1.783544	2.41E-11	1.36E-10
UCN2	0.72836626	4.120332153	2.500025	1.80E-19	6.73E-18
VEGFC	2.4144167	10.2701672	2.088713	3.42E-08	1.14E-07
VGF	0.14444269	0.441491762	1.611889	5.04E-08	1.63E-07
ACVR1C	0.18085289	0.522859525	1.531607	9.15E-13	6.62E-12
ANGPTL1	4.0469674	0.423402202	−3.25674	1.11E-17	2.51E-16
AR	0.84142851	0.279458225	−1.59021	2.28E-13	1.84E-12
BMPR1B	0.42433224	0.97757387	1.204011	3.72E-07	1.05E-06
CNTFR	3.5781603	0.853604306	−2.06758	2.78E-14	2.71E-13
EGFR	15.5484376	39.46628352	1.343851	5.59E-07	1.54E-06
EPOR	0.4418559	1.051799214	1.251211	9.80E-15	1.03E-13
FGFR4	0.40046898	1.471692526	1.877714	3.35E-12	2.20E-11
IGF1R	4.97994875	9.993087189	1.0048	7.96E-12	4.88E-11
IL12RB1	0.52176071	1.430100231	1.454656	1.35E-07	4.07E-07
IL12RB2	0.55232543	2.540589041	2.201573	8.83E-09	3.25E-08
IL15RA	2.65784681	5.826970161	1.132488	1.44E-12	1.00E-11
IL17RD	1.96227708	0.814741728	−1.26811	2.67E-05	5.67E-05
IL21R	0.32308714	1.142605044	1.822332	6.22E-09	2.35E-08
IL22RA2	0.1338516	0.453174929	1.759434	6.36E-07	1.74E-06
IL27RA	3.5496236	9.703778415	1.450881	8.17E-09	3.03E-08
IL2RA	0.74667359	2.439247542	1.707887	7.69E-12	4.72E-11
IL2RG	5.99842991	13.36207169	1.155487	0.002085	0.003336
IL31RA	0.28666596	0.791027258	1.464357	4.68E-07	1.31E-06
LEPR	2.10085142	0.903359942	−1.2176	6.96E-06	1.62E-05
LGR5	0.56930049	1.348318763	1.243899	0.002398	0.0038
MC1R	0.22552167	0.629522882	1.480993	9.02E-16	1.22E-14
MET	6.4272196	16.07767268	1.322792	2.07E-13	1.69E-12
NR2E1	0.06145965	0.268358343	2.126449	0.00037	0.000665
NR3C2	2.06675998	0.367105007	−2.49311	9.20E-15	9.80E-14
NR4A1	23.7674383	7.62137528	−1.64086	6.87E-06	1.60E-05
NR4A3	4.13466038	1.581359678	−1.3866	0.001584	0.002583
NR5A1	0.01405336	1.762426165	6.970504	8.63E-07	2.31E-06
PTGER3	1.01346032	0.389626298	−1.37913	3.12E-07	8.95E-07
PTGFR	1.32754176	0.439742298	−1.59403	2.32E-05	4.98E-05
RORC	4.24330743	0.607971833	−2.80311	2.66E-21	1.90E-19
RXRG	0.48690854	0.202918239	−1.26275	5.87E-06	1.38E-05
SORT1	18.0520794	8.066695852	−1.16212	8.58E-18	2.00E-16
SSTR2	0.10550506	0.406339649	1.945374	4.01E-08	1.32E-07
TACR1	0.68462766	0.172061105	−1.9924	2.36E-15	2.91E-14
TGFBR3	4.78706853	1.379474384	−1.79502	5.43E-14	5.01E-13
TNFRSF11A	1.49016855	0.523554639	−1.50906	4.12E-12	2.66E-11
TNFRSF12A	17.7723627	56.19964292	1.660925	4.14E-15	4.85E-14
TNFRSF18	4.3003038	16.11444457	1.905844	5.18E-11	2.73E-10
TNFRSF19	6.77463912	2.906295251	−1.22096	3.60E-08	1.19E-07
TNFRSF25	1.76759089	4.574161764	1.371723	4.06E-12	2.62E-11
TNFRSF4	0.74157962	3.237799059	2.12634	4.82E-18	1.21E-16
TNFRSF8	0.23116456	0.716018319	1.631076	3.15E-10	1.48E-09
TNFRSF9	0.21987028	1.011939319	2.202398	2.09E-15	2.60E-14
TUBB3	0.35949321	1.485323276	2.046741	4.84E-16	6.97E-15
FCGR3A	3.9329154	14.23552539	1.855825	6.68E-11	3.46E-10
FCGR3B	0.33728536	0.76681787	1.184914	0.000267	0.000491
CD247	0.83658503	1.750058596	1.064819	0.000918	0.001553
ZAP70	0.650674	1.400126297	1.10555	0.000721	0.001239
SHC1	21.3436652	43.46714575	1.026117	5.53E-17	1.00E-15
SH2D1B	1.30987534	0.248778859	−2.39649	0.000128	0.000245
SH2D1A	0.48679012	1.030303921	1.081698	0.01699	0.023428
GZMB	3.38306871	9.73932144	1.525489	1.80E-07	5.36E-07
PRF1	2.17381804	6.330644293	1.542121	1.15E-06	3.03E-06
BID	3.94088989	9.343639602	1.245463	6.89E-16	9.59E-15
TEC	1.46982345	0.60201631	−1.28777	6.31E-13	4.69E-12
ICOS	0.44116172	1.427023302	1.693629	6.12E-11	3.19E-10
CTLA4	0.50396065	2.170800093	2.106844	2.15E-14	2.15E-13
CBLB	1.28623151	3.026043292	1.234282	8.12E-16	1.11E-14
CDK4	14.1156616	28.71289684	1.024402	1.04E-18	3.14E-17
PDK1	1.06635448	2.243918541	1.073333	1.46E-11	8.57E-11
TRAV2	0.22091724	0.51174568	1.211921	0.005292	0.007947
TRAV4	0.22383663	0.516858401	1.207323	0.003459	0.005347
TRAV8-3	0.24255669	0.56073964	1.209009	0.003018	0.004704
TRAV8-4	0.18845464	0.418056464	1.149481	0.008897	0.012887
TRAV8-6	0.22023349	0.467775712	1.086783	0.004075	0.006228
TRAV24	0.08147574	0.226358104	1.474164	0.02406	0.032283
TRAV26-1	0.14875179	0.338231849	1.185105	0.000173	0.000326
TRAV26-2	0.10735569	0.241319312	1.168545	0.008334	0.01214
TRAV29DV5	0.14335924	0.3344734	1.222257	0.00784	0.011471
TRBJ2-3	0.36593755	0.882313743	1.269694	0.005042	0.007595
TRBV4-1	0.18012141	0.478476686	1.409479	0.020608	0.027965

The FDR means the false discovery rate and the FDR value is less than 0.05 as the filter criterion. Tumor mean represented the average gene expression in tumor samples and normal mean represented the average gene expression in normal samples.

### Identification of survival-associated IRGs

**Figure 3:**
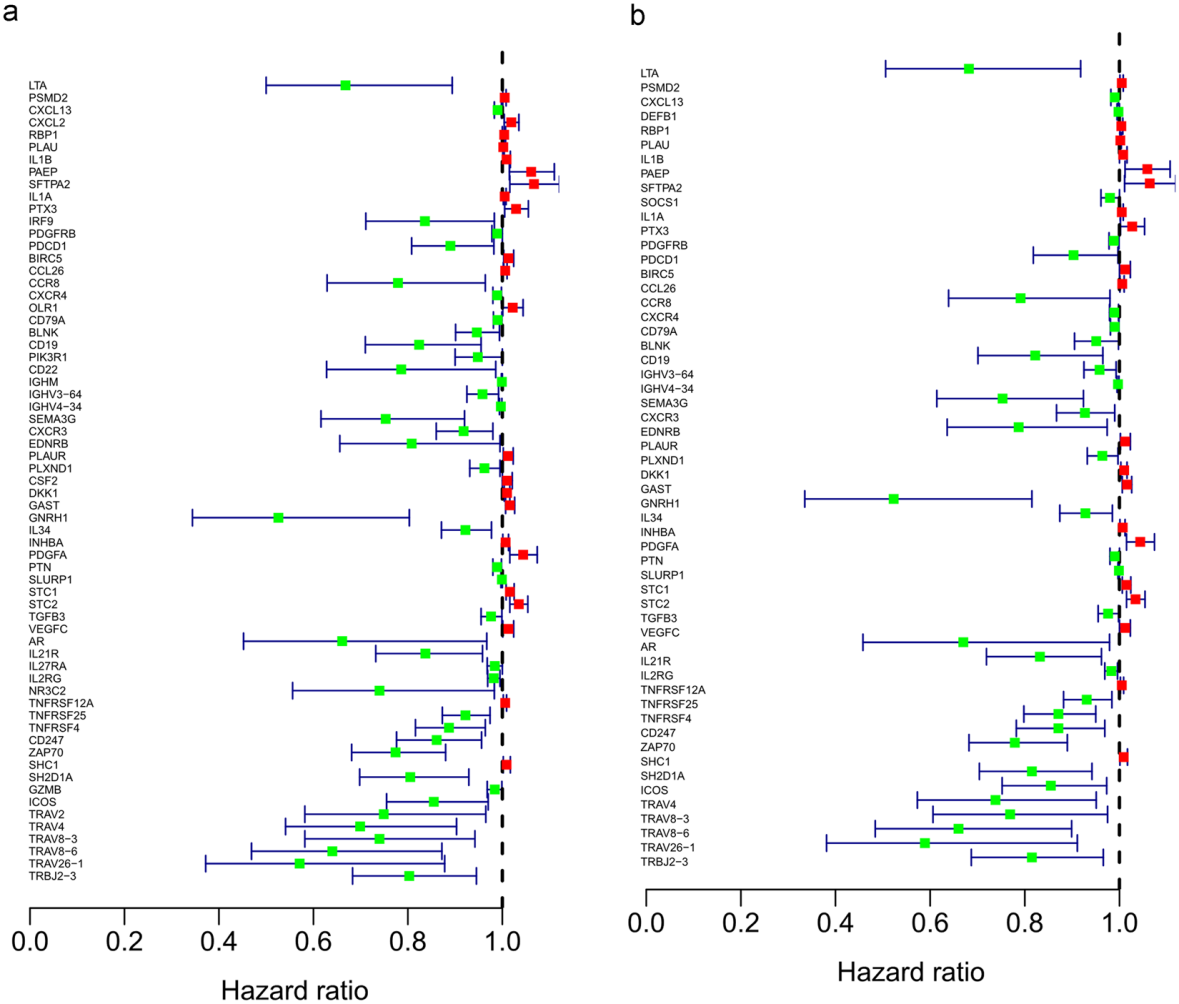
Forest plot of the hazard ratios showing the prognosis-related immune genes. (**a**) The prognosis-related genes in total HNSCC patients, (**b**) The prognosis-related genes in HPV- HNSCC patients. Red dots represent high-risk genes (HR > 1), and green dots represent low-risk genes (HR < 1).

**Figure 4:**
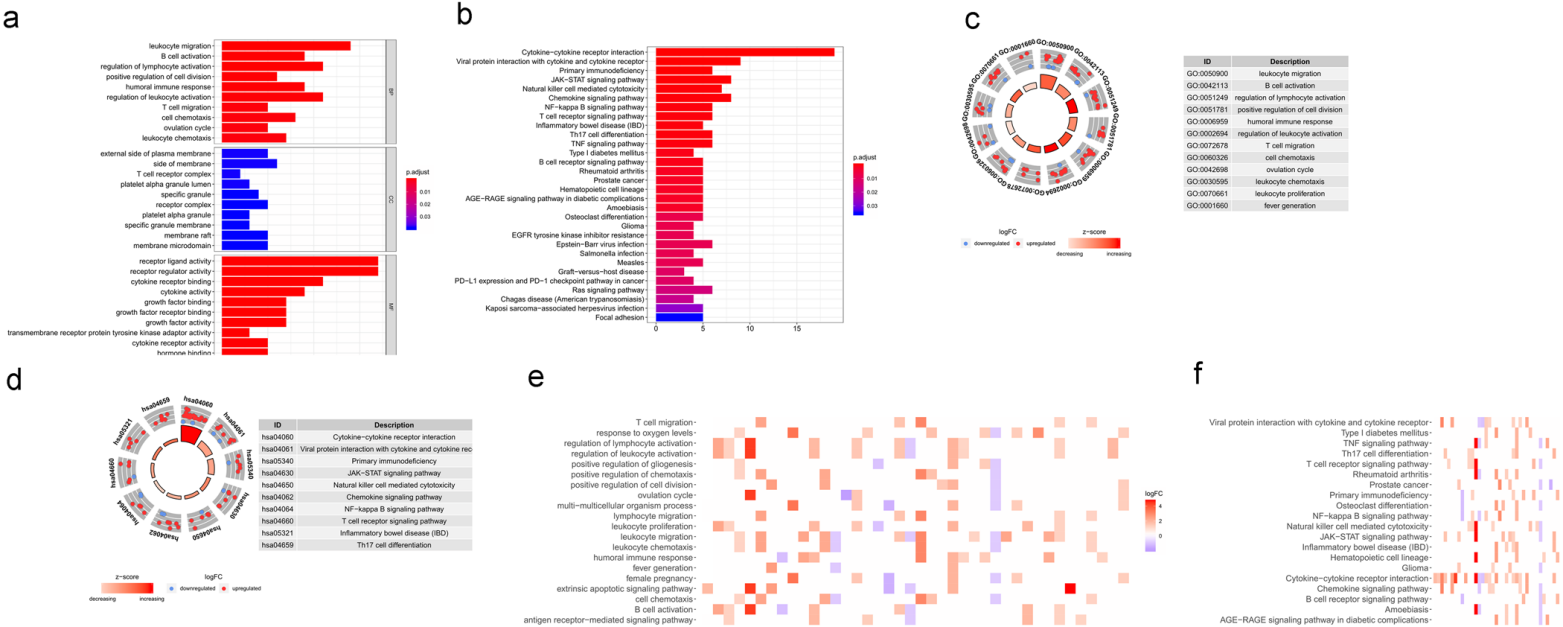
Functional enrichment of survival-associated IRGs. Survival-associated IRGs identified via Gene Ontology (GO) analysis (**a**) and KEGG pathway analysis (**b**). Circle graphs representing the most significant Gene Ontology terms (**c**) and Kyoto Encyclopedia of Genes and Genomes pathways (**d**). Nodes in the concentric circle graph represent co-expressed genes clustered in specific biological process terms. The inner sectors with larger size and darker color represent more significant enrichment. Heat maps representing the overall expression levels of 20 enriched Gene Ontology terms (**e**) and Kyoto Encyclopedia of Genes and Genomes pathways (**f**).

**Figure 5:**
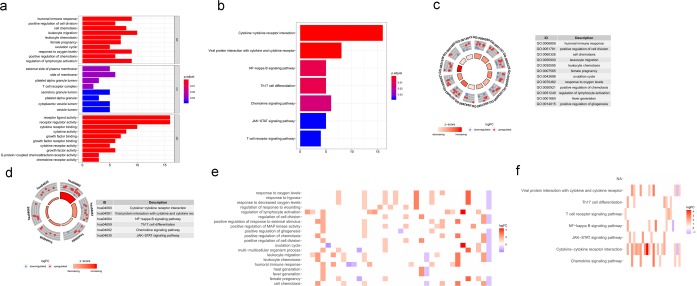
Functional enrichment of HPV- HNSCC patients survival-associated IRGs. Survival-associated IRGs identified via Gene Ontology (GO) analysis (**a**) and KEGG pathway analysis (**b**). Circle graphs representing the most significant Gene Ontology terms (**c**) and Kyoto Encyclopedia of Genes and Genomes pathways (**d**). Nodes in the concentric circle graph represent co-expressed genes clustered in specific biological process terms. The inner sectors with larger size and darker color represent more significant enrichment. Heat maps representing the overall expression levels of 20 enriched Gene Ontology terms (**e**) and 7 enriched Kyoto Encyclopedia of Genes and Genomes pathways (**f**).

To explore the relationship between IRGs and prognosis, we identified 65, 56 IRGs that were significantly associated with overall survival (OS) from the total and HPV- HNSCC patients, respectively (P < 0.05; Table 2A,B; Fig. 3a,b). Moreover, we divided 65 IRGs into 23 high-risk (HR > 1) genes and 42 low-risk (HR < 1) genes (Fig. 3a). We also identified 20 high-risk (HR > 1) genes and 36 low-risk (HR < 1) genes from 56 IRGs (Fig. 3b). Similar to the results from the previous enrichment analysis of differentially expressed IRGs, 65 survival-associated IRGs were mainly involved in several Gene Ontology (GO) terms related to immunocyte migration and the activity of receptors and ligands (Fig. 4a,c,e). Additionally, KEGG pathway analysis indicated that survival-associated IRGs were enriched in “cytokine-cytokine receptor interaction (hsa04060)” (Fig. 4b,d,f). Interestingly, the results of 56 survival-associated IRGs enrichment analysis were almost consistent with our earlier research. GO terms immunocyte migration and the activity of receptors and ligands were mainly enriched (Fig. 5a,c,e). KEGG pathway “cytokine-cytokine receptor interaction (hsa04060)” was also enriched (Fig. 5b,d,f).

**Table 2:** Relationships between the expression of immune-related genes and overall survival in head and neck squamous cell carcinoma.

Gene	HR	HR95%CI	HR95%CI	P value
LTA	0.668496	0.499696	0.894317	0.006684
PSMD2	1.004766	1.001218	1.008327	0.008437
CXCL13	0.990465	0.982905	0.998082	0.014244
CXCL2	1.019352	1.003773	1.035172	0.014722
RBP1	1.003664	1.000355	1.006985	0.029978
PLAU	1.0024	1.000922	1.00388	0.001448
IL1B	1.008894	1.001188	1.01666	0.023602
PAEP	1.061454	1.015316	1.10969	0.00853
SFTPA2	1.067483	1.016221	1.12133	0.0093
IL1A	1.005049	1.002307	1.007798	0.000302
PTX3	1.029444	1.004951	1.054535	0.018179
IRF9	0.835539	0.710554	0.982507	0.029748
PDGFRB	0.9891	0.978489	0.999825	0.046403
PDCD1	0.890434	0.807518	0.981864	0.019967
BIRC5	1.013461	1.002572	1.024469	0.015264
CCL26	1.005943	1.001596	1.010309	0.00733
CCR8	0.778939	0.629352	0.96408	0.021665
CXCR4	0.98885	0.980251	0.997524	0.011864
OLR1	1.021934	1.000659	1.043662	0.043248
CD79A	0.990108	0.981287	0.999008	0.02945
BLNK	0.946274	0.90121	0.993591	0.026538
CD19	0.823527	0.710098	0.955076	0.010232
PIK3R1	0.948401	0.899575	0.999877	0.04947
CD22	0.786499	0.627544	0.985716	0.037082
IGHM	0.998821	0.99779	0.999852	0.025075
IGHV3-64	0.957713	0.924915	0.991675	0.015091
IGHV4-34	0.99686	0.99438	0.999347	0.013377
SEMA3G	0.753086	0.616466	0.919983	0.005494
CXCR3	0.918157	0.860136	0.980092	0.010355
EDNRB	0.807825	0.65587	0.994985	0.044723
PLAUR	1.012167	1.001904	1.022535	0.02003
PLXND1	0.96239	0.93088	0.994967	0.024004
CSF2	1.010432	1.000419	1.020545	0.041111
DKK1	1.009998	1.003671	1.016365	0.001918
GAST	1.016163	1.006507	1.025912	0.000997
GNRH1	0.525898	0.344358	0.803142	0.002933
IL34	0.922265	0.870564	0.977036	0.005974
INHBA	1.007063	1.00146	1.012697	0.013411
PDGFA	1.044258	1.015614	1.073709	0.002275
PTN	0.988966	0.979748	0.998271	0.02022
SLURP1	0.99868	0.997466	0.999896	0.033426
STC1	1.016164	1.00751	1.024892	0.000238
STC2	1.034862	1.015672	1.054415	0.000333
TGFB3	0.976459	0.95481	0.9986	0.037301
VEGFC	1.012665	1.00125	1.024211	0.02956
AR	0.660928	0.451694	0.967084	0.032982
IL21R	0.83736	0.732213	0.957607	0.009523
IL27RA	0.983581	0.967608	0.999818	0.047502
IL2RG	0.981909	0.968983	0.995008	0.006932
NR3C2	0.739584	0.55636	0.983148	0.037804
TNFRSF12A	1.005603	1.001962	1.009258	0.002538
TNFRSF25	0.92232	0.873419	0.97396	0.003623
TNFRSF4	0.887354	0.816476	0.964384	0.004896
CD247	0.861279	0.775609	0.956411	0.005211
ZAP70	0.773955	0.680878	0.879756	8.87E-05
SHC1	1.009126	1.001624	1.016684	0.017021
SH2D1A	0.805145	0.697566	0.929315	0.003059
GZMB	0.983635	0.968192	0.999325	0.040998
ICOS	0.855448	0.754597	0.969777	0.014709
TRAV2	0.749408	0.581806	0.965292	0.025518
TRAV4	0.698698	0.540568	0.903086	0.00617
TRAV8-3	0.739954	0.581527	0.941541	0.014286
TRAV8-6	0.639837	0.46938	0.872197	0.004727
TRAV26-1	0.571343	0.371677	0.87827	0.010721
TRBJ2-3	0.803312	0.682749	0.945165	0.008298
**Gene**	**HR**	**HR95%CI**	**HR95%CI**	**P value**
LTA	0.681712	0.506441	0.917639	0.011511
PSMD2	1.004734	1.001177	1.008303	0.009049
CXCL13	0.989825	0.981869	0.997845	0.012991
DEFB1	0.997616	0.995385	0.999852	0.036633
RBP1	1.004001	1.000537	1.007476	0.023552
PLAU	1.002342	1.000849	1.003836	0.002091
IL1B	1.008056	1.000107	1.016068	0.046975
PAEP	1.058761	1.012306	1.107348	0.012622
SFTPA2	1.063654	1.010685	1.119399	0.017896
SOCS1	0.98023	0.961002	0.999843	0.048214
IL1A	1.004939	1.002162	1.007723	0.000483
PTX3	1.027288	1.002224	1.05298	0.032662
PDGFRB	0.988423	0.977725	0.999239	0.035982
PDCD1	0.903214	0.817886	0.997445	0.04438
BIRC5	1.011754	1.000279	1.02336	0.044647
CCL26	1.005815	1.001453	1.010196	0.008926
CCR8	0.791404	0.639114	0.979982	0.031921
CXCR4	0.988743	0.979416	0.998159	0.019232
CD79A	0.989859	0.980521	0.999287	0.035085
BLNK	0.950602	0.905354	0.998112	0.041761
CD19	0.822477	0.701205	0.964723	0.01634
IGHV3-64	0.958206	0.925079	0.992519	0.017394
IGHV4-34	0.997012	0.994536	0.999494	0.018331
SEMA3G	0.753482	0.614441	0.923987	0.006537
CXCR3	0.92655	0.867316	0.989829	0.023621
EDNRB	0.787011	0.635774	0.974225	0.027821
PLAUR	1.012231	1.002034	1.022531	0.018602
PLXND1	0.963663	0.93165	0.996776	0.03177
DKK1	1.0097	1.003305	1.016136	0.002901
GAST	1.015899	1.006204	1.025687	0.001263
GNRH1	0.522748	0.335136	0.815388	0.00424
IL34	0.927659	0.873885	0.984741	0.013715
INHBA	1.006638	1.000945	1.012363	0.022231
PDGFA	1.043827	1.014846	1.073636	0.002829
PTN	0.990012	0.980399	0.999719	0.043749
SLURP1	0.998546	0.997284	0.999809	0.024046
STC1	1.015356	1.006415	1.024377	0.000733
STC2	1.034271	1.015038	1.053869	0.000434
TGFB3	0.976175	0.954551	0.998288	0.034869
VEGFC	1.011847	1.00034	1.023486	0.043569
AR	0.669584	0.457741	0.979467	0.038746
IL21R	0.831783	0.719299	0.961857	0.012971
IL2RG	0.982533	0.968926	0.99633	0.013261
TNFRSF12A	1.005413	1.001712	1.009127	0.004113
TNFRSF25	0.931332	0.881721	0.983734	0.01086
TNFRSF4	0.870867	0.798383	0.949931	0.001818
CD247	0.870532	0.78222	0.968814	0.01107
ZAP70	0.779149	0.68226	0.889798	0.00023
SHC1	1.009006	1.001361	1.016708	0.02086
SH2D1A	0.814511	0.704142	0.942179	0.005751
ICOS	0.855312	0.752063	0.972737	0.017261
TRAV4	0.738136	0.573219	0.9505	0.018599
TRAV8-3	0.76872	0.605943	0.975226	0.030265
TRAV8-6	0.659702	0.483854	0.89946	0.008541
TRAV26-1	0.589333	0.381387	0.910658	0.017244
TRBJ2-3	0.81451	0.68679	0.965983	0.01839

(A) The prognosis-related genes in total HNSCC patients, (B) The prognosis-related genes in HPV- HNSCC patients. Immune-related genes were divided into high risk and low risk the prognosis-related genes via HR value (HR > 1 indicated high risk and HR < 1 indicated low risk).

### Transcription factor (TF) regulatory network

**Figure 6:**
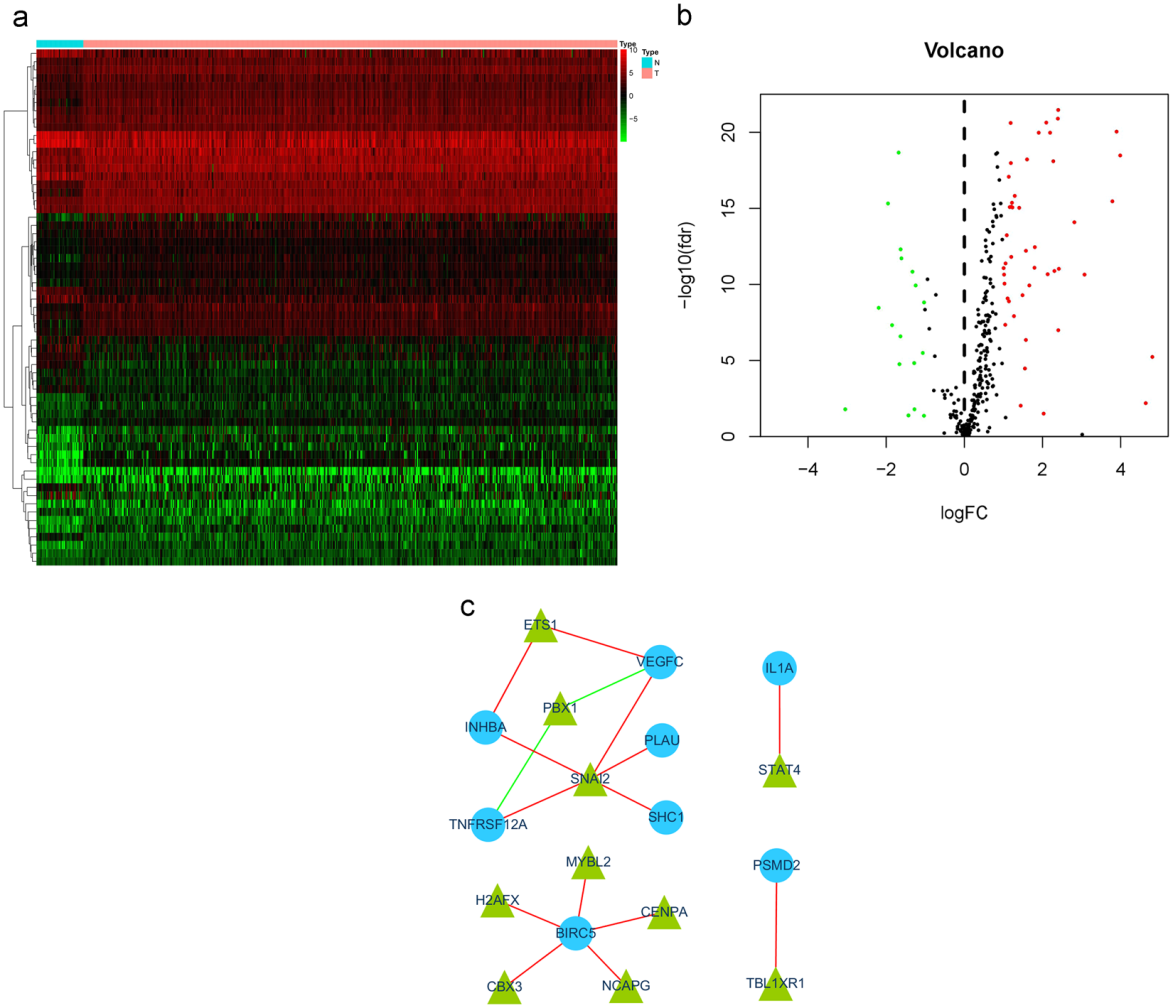
Differentially expressed transcription factors (TFs) in the differentially expressed genes between HNSCC and nontumor patients. (**a**) Heat map and (**b**) volcano plot demonstrating differentially expressed TFs. Red represents upregulated differentially expressed TFs, blue represents downregulated differentially expressed TFs, and black dots (volcano) represent TFs that were not differentially expressed. **(c**) Differentially expressed TFs and the high-risk survival-associated IRG interaction network. The red line represents positive regulation, and the green line represents negative regulation.

We found 63 differentially expressed transcription factors (TFs) within the genes that were differentially expressed between HNSCC patients and normal patients, 46 of which were upregulated and 17 of which were downregulated (Fig. 6a,b). To investigate the relationship between the differentially expressed TFs and 23 high-risk survival-associated IRGs, we constructed a regulatory network based on them. In this module (Fig. 6c), 16 edges involving 18 nodes were formed. SNA12 was remarkable for having the most connections with other high-risk genes, while BIRC5 was remarkable for having the most connections with other TFs.

### Immune gene-related prognosis model

To establish a prognosis prediction model based on survival-associated IRG expression, prognostic genes for HNSCC were identified by multivariable Cox regression analysis. Finally, to avoid false positive results, 10 genes were proven to be predictive of clinical outcomes via multivariable Cox regression analysis (P < 0.01) and constructed the optimal model according to Akaike information criterion. The model corresponding to minimum AIC value (AIC = 1,859.31) represented the target model. SEMA3G, GNRH1 and ZAP70 were positively correlated with OS. PLAU, SFTPA2, CCL26, DKK1, GAST, PDGFA and STC1 were negatively correlated with OS (Table 3A). Thus, the expression data of these prognostic genes and their coefficients were used to develop a gene-based prognosis prediction model, with a formula as follows: [expression level of PLAU * (0.0013)] + [expression level of SFTPA2 * (0.0590)] + [expression level of CCL26 * (0.0081)] + [expression level of SEMA3G * (−0.1523)] + [expression level of DKK1 * (0.0059)] + [expression level of GAST * (0.0173) + [expression

**Table 3:** The coefficients and HR values of prognosis-associated IRGs included in the prognosis prediction model.

Gene	coef	HR	HR95%CI	HR95%CI	P value
PLAU	0.001272	1.001273	0.999585	1.002964	0.139493
SFTPA2	0.059045	1.060823	1.008968	1.115344	0.020938
CCL26	0.008102	1.008135	1.003507	1.012784	0.000558
SEMA3G	−0.15228	0.858744	0.705155	1.045787	0.129856
DKK1	0.005884	1.005902	0.998694	1.013162	0.108773
GAST	0.017251	1.0174	1.007052	1.027855	0.000942
GNRH1	−0.47171	0.623937	0.389605	0.99921	0.049617
PDGFA	0.026347	1.026697	0.994729	1.059693	0.102572
STC1	0.01324	1.013328	1.003745	1.023003	0.006312
ZAP70	−0.12972	0.878341	0.758798	1.016718	0.082239
**Gene**	**coef**	**HR**	**HR95%CI**	**HR95%CI**	**P value**
PLAU	0.001459	1.00146	0.999768	1.003154	0.090764
CCL26	0.008962	1.009002	1.004356	1.013669	0.000141
SEMA3G	−0.15543	0.856043	0.694721	1.054827	0.144578
DKK1	0.005887	1.005904	0.99853	1.013332	0.116845
GAST	0.017418	1.017571	1.007484	1.027759	0.000611
GNRH1	−0.53635	0.584881	0.359709	0.951008	0.03058
PDGFA	0.03171	1.032218	1.000327	1.065127	0.047663
STC1	0.011712	1.011781	1.001787	1.021874	0.02075
TNFRSF4	−0.07703	0.925858	0.829668	1.0332	0.168698
ZAP70	−0.18953	0.827345	0.645459	1.060486	0.134569
SH2D1A	0.174511	1.190664	0.947321	1.496515	0.134644

(A) Total HNSCC patients, (B) HPV- HNSCC patients.

level of GNRH1 * (−0.4717)] + [expression level of PDGFA * (0.0263)] + [expression level of STC1 * (0.0132)] + [expression level of ZAP70 * (−0.1297)]. The 10-gene-based model was used to calculate a risk score for each sample as described above (Fig. 7a,c,e). All patients were divided into a high-risk group (n = 237) or a low-risk group (n = 238) according to the median risk score after removing some patients with missing clinical characteristics data. According to the Kaplan-Meier survival analysis, the overall survival time was significantly different between the high-risk and low-risk groups, and the five-year survival rates were 31.2% and 66.8%, respectively (Fig. 8a). The area under the curve (AUC) of the receiver operating characteristic (ROC) curve was 0.750, suggesting that the model could predict the survival outcomes of HNSCC patients (Fig. 8c).

**Figure 7:**
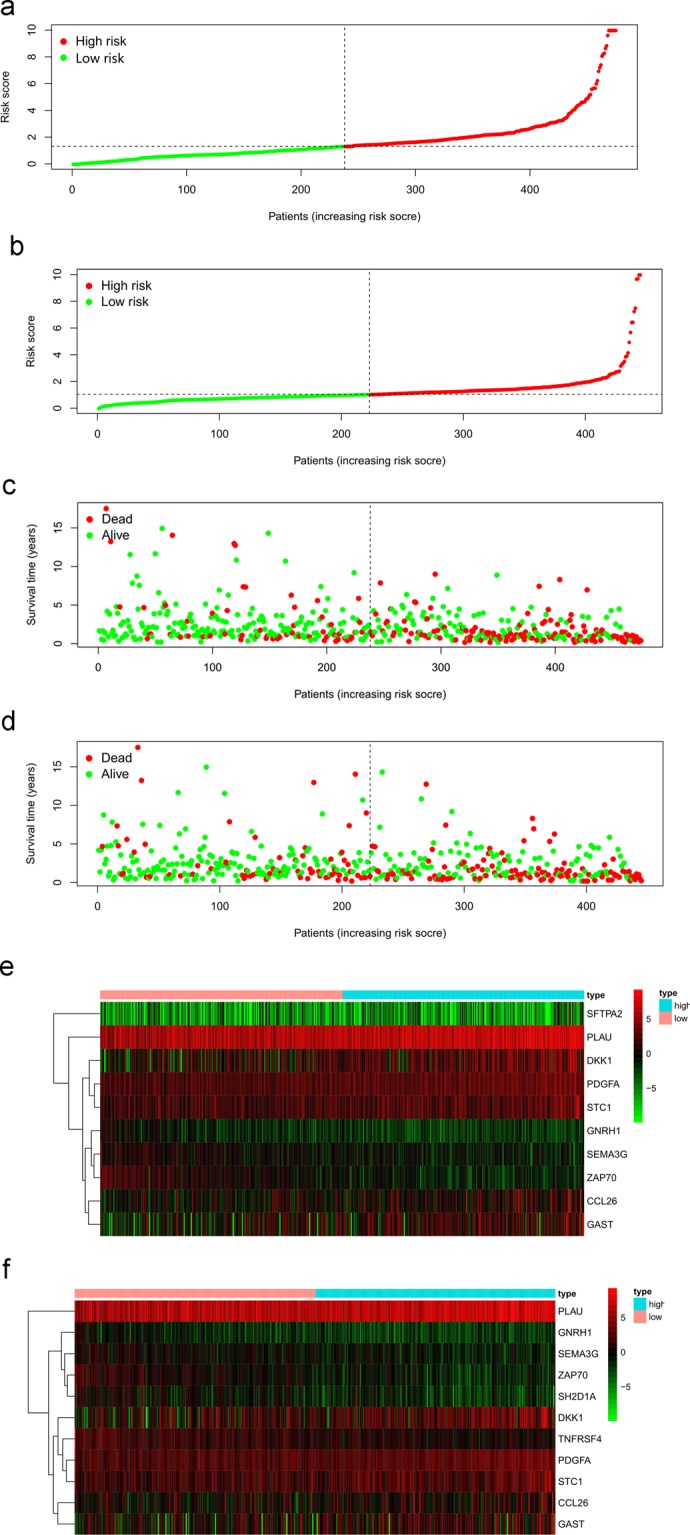
Development of a risk score based on survival-associated genes in total HNSCC patients (**a,c,e**) and HPV- HNSCC patients (**b,d,f**). (**a,b**) Association between risk score and the distribution of high (red) and low (green) risk groups. (**c,d**) Survival status of the HNSCC patients in different groups; red and green dots represent deceased and surviving patients, respectively. (**e**,**f**) Heat map representing the expression level of the prognostic genes based on the high-risk group and the low-risk group.

**Figure 8:**
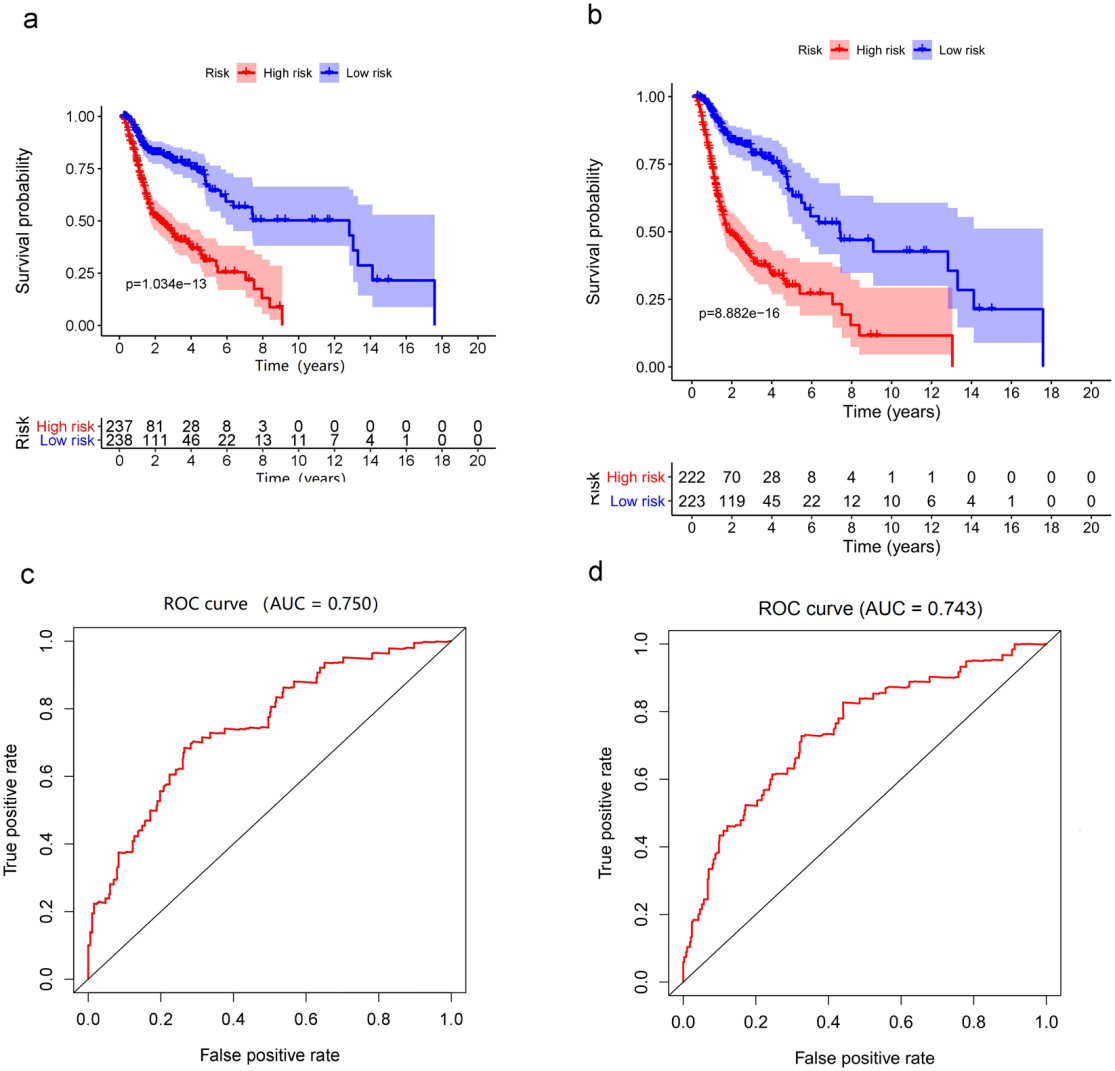
Survival analysis in different groups of HNSCC patients. (**a**) The overall survival rates of different groups of HNSCC patients. (**b**) The overall survival rates of different groups of HPV-HNSCC patients. Verification of the accuracy of the prognostic model via analysis of the area under curve (AUC) of the receiver operating characteristic (ROC) curve (**c)**. Total HNSCC patients, (**d**) HPV- HNSCC patients).

For HPV- HNSCC patients, we also identified 11 genes to predict clinical outcomes and establish the prognosis predict model via multivariable Cox regression analysis (P < 0.01). The same as the above methods and standards, the model corresponding to minimum AIC value (AIC = 1814.05) represented the optimal model. SEMA3G, GNRH1, TNFRSF4 and ZAP70 were positively correlated with OS. PLAU, SH2D1A, CCL26, DKK1, GAST, PDGFA and STC1 were negatively correlated with OS (Table 3B). Then, the 11-gene-based model was also used to calculate a risk score for each sample with an alike formula (Fig. 7b,d,f). Similarly, HPV- patients were divided into a high-risk group (n = 222) or a low-risk group (n = 223) and the five-year survival rates were 28.5% and 63.1%, respectively (Fig. 8b). The area under the curve (AUC) of the receiver operating characteristic (ROC) curve was 0.743, suggesting that the model could predict the survival outcomes of HPV- HNSCC patients (Fig. 8d).

### The relationship between IRGs and clinical factors

**Figure 9:**
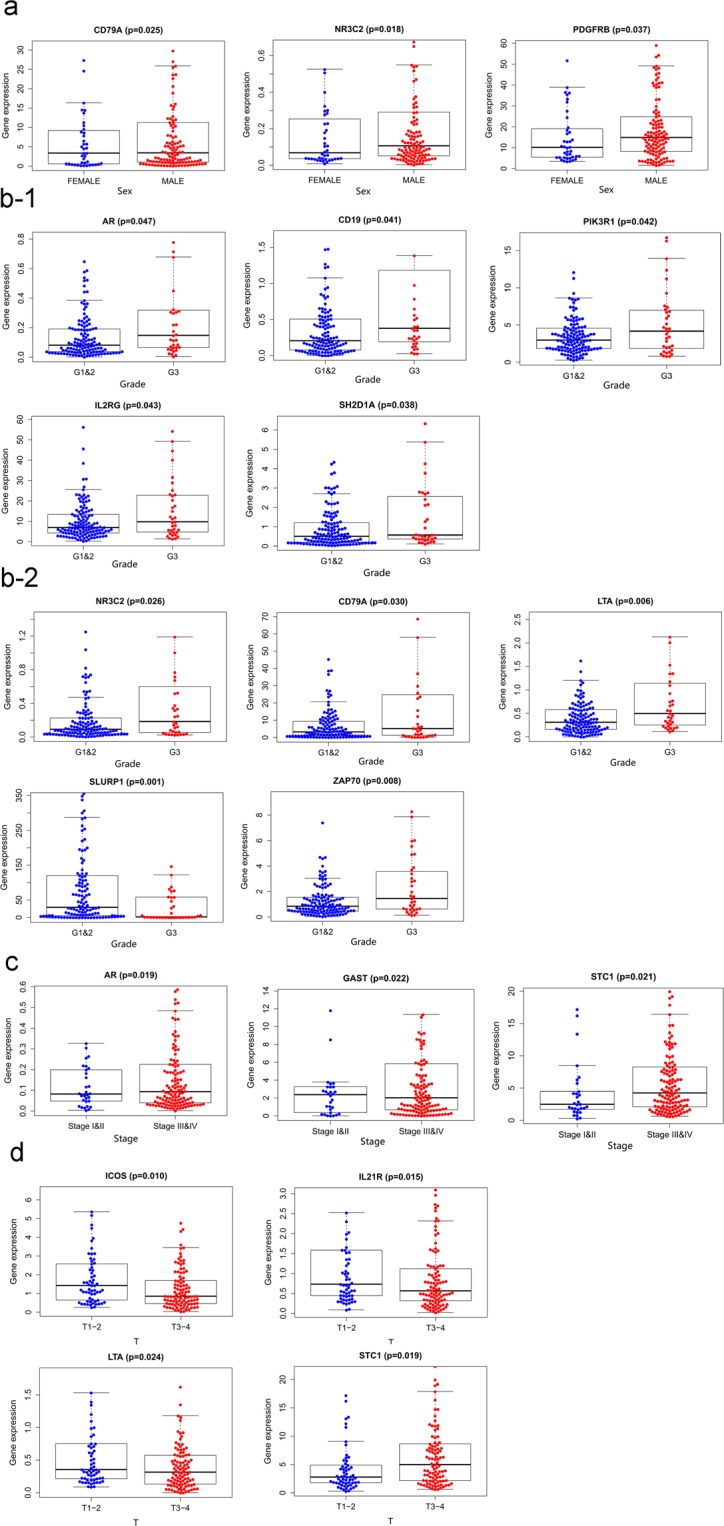
The relationships between prognosis-related IRG expression and sex (**a**), tumor grade (**b**), tumor stage (**c**), and T stage (**d**) in the high-risk (red) and low-risk (blue) groups of total HNSCC patients. The relationships between the expression of genes (SLURP1, ZAP70, CD79A, LTA, and NR3C2) (B2) and tumor grade were more significant than AR, CD19, IL2RG, PIK3R1 and SH2D1A (B1) according to p value.

**Figure 10:**
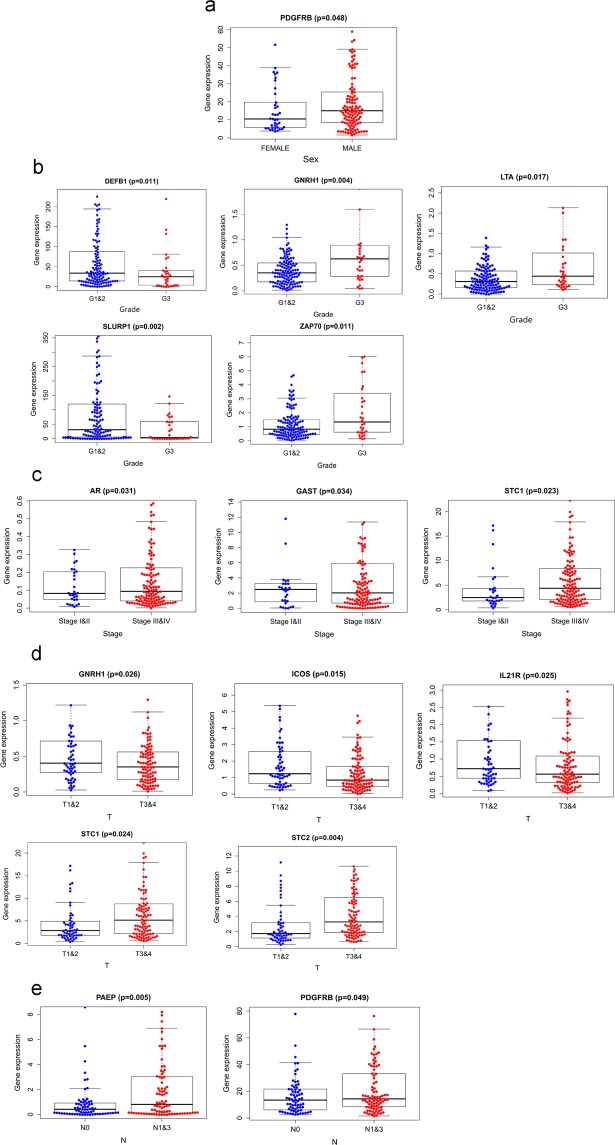
The relationships between prognosis-related IRG expression and sex (**a**), tumor grade (**b**), tumor stage (**c**), T stage (**d**) and N stage (**e**) in the high-risk (red) and low-risk (blue) groups of the HPV- HNSCC patients.

The relationships between the single genes and clinical factors were analyzed. The results indicated that the gene expression level of PDGFRB was significantly higher in males than in females whether total HNSCC patients (Fig. 9a) or HPV- HNSCC patients (Fig. 10a). AR was significantly higher in stage III and IV disease than in stage I and II disease, ICOS was significantly higher in T1–2 disease than in T3–4 disease whether total HNSCC patients (Fig. 9c,d) or HPV- HNSCC patients (Fig. 10c,d). Among the total HNSCC patients, NR3C2 was significantly higher in grade 1 and 2 disease than in grade 3 disease (Fig. 9b). Among the HPV- HNSCC patients, DEFB1 was significantly higher in grade 1 and 2 disease than in grade 3 disease, PAEP was significantly higher in N0 disease than in N1–3 disease (Fig. 10b,e).

### The relevance analysis of risk score and immune cell infiltration

**Figure 11:**
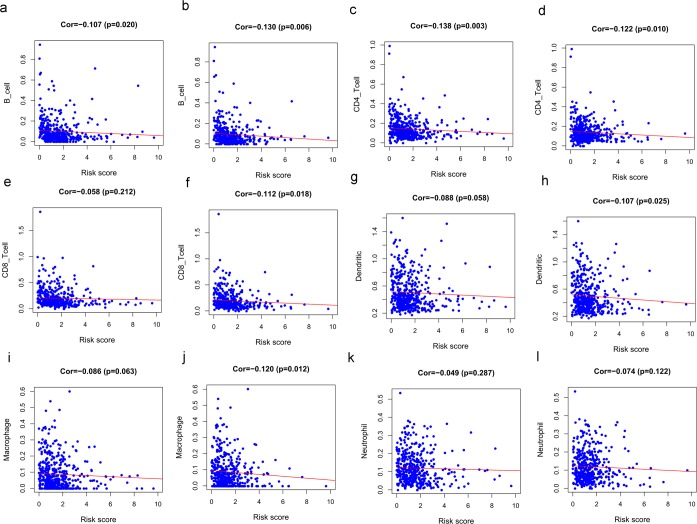
The relationships between infiltration abundances of six types of immune cells and the risk score in different groups of HNSCC patients. B cell and CD4 T cell infiltration levels were significantly negatively correlated with the risk score in both total HNSCC patients (**a,c**) and HPV- patients (**b,d**). In total HNSCC patients, the infiltration of CD8 T cells (**e**), dendritic cells (**g**) and macrophages (**i**) were not statistically significant (P > 0.05) while CD8 T cells (**f**), dendritic cells (**h**) and macrophages (**j**) infiltration levels were significantly negatively correlated with the risk score of the HPV- patients. The infiltration of neutrophils was not statistically significant (P > 0.05) in both total (**k**) and HPV- (**l**) HNSCC patients.

To reveal whether the immune-related genome can alter the tumor immune microenvironment, we analyzed the relationship between the risk score of IRGs and immune cell infiltration. B cell and CD4 T cell infiltration levels were significantly negatively correlated with the risk score in both total HNSCC patients (Fig. 11a,c) and HPV- patients (Fig. 11b,d) (P < 0.05). In total HNSCC patients, the infiltration of CD8 T cells, dendritic cells and macrophages were not statistically significant (P > 0.05) (Fig. 11e,g,i), while CD8 T cells, dendritic cells and macrophages infiltration levels were significantly negatively correlated with the risk score in the HPV- patients (P < 0.05) (Fig. 11f,h,j). The infiltration of neutrophils was not statistically significant in both total and HPV- HNSCC patients (P > 0.05) (Fig. 11k,l).

### Independent survival analysis

**Figure 12:**
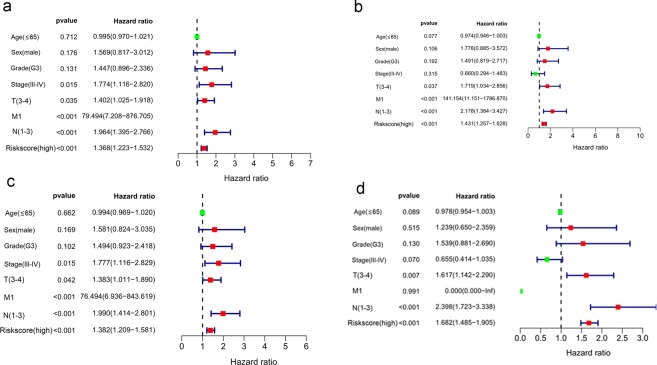
Univariable (**a,c**) and multivariable (**b,d**) independent subgroup analysis in terms of overall survival for the total HNSCC patients (**a,b**) and HPV- HNSCC patients (**c,d**).

The univariable and multivariable Cox regression analyses suggested that the risk score could become an independent predictor for OS after adjusting for other parameters, including TN stage and risk score whether total HNSCC patients (Fig. 12a,b) or HPV- HNSCC patients (Fig. 12c,d) (P < 0.05).

### Validation in the oncomine database

**Figure 13:**
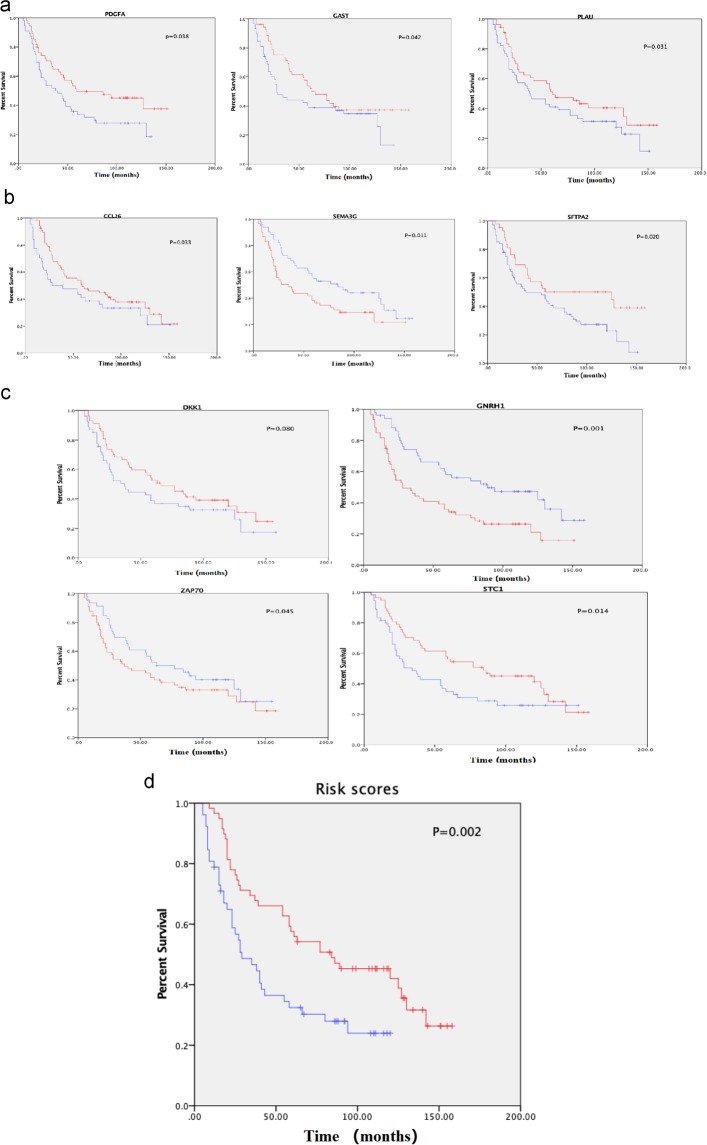
Validation of TCGA results with Oncomine data. Kaplan-Meier survival curves were used to verify the relationship between genes (**a**). PDGFA, GAST and PLAU; (**b**) CCL26, SEMA3G and SFTPA2; (**c**) DKK1, GNRH1, ZAP70 and STC1) and risk scores (**d**) with overall survival. Red represents the high-risk group, and blue represents the low-risk group.

To verify the above results, we selected available cohorts of HNSCC samples from the Oncomine database, including Rickman’s cohort (81 samples) and Cormer’s cohort (31 samples). We extracted the expression levels of the survival-related IRGs, relevant clinical characteristics and follow-up times, and we performed survival analysis and created Kaplan-Meier survival curves (Fig. 13). As a result, 10 of the survival-related IRGs we identified were significantly associated with clinical prognosis according to the survival curves. SEMA3G, GNRH1 and ZAP70 were negatively correlated with OS, whereas PLAU, SFTPA2, CCL26, DKK1, GAST, PDGFA and STC1 were positively correlated with OS. Similarly, we also calculated the risk scores of 112 samples and created survival curves (Fig. 13). The results from this verification cohort were consistent with the results we obtained before, indicating their reliability and repeatability.

## Discussion

The head and neck squamous cell carcinoma (HNSCC) is a common cancer which has attracted considerable and increasing attention^12^. While many patients with locally advanced HNSCC could be treated by surgery, radiation, chemotherapy and the combination application, those who develop recurrent/metastatic disease (R/M) has a median overall survival of less than a year^13^. Therefore, because second-line treatment options for advanced HNSCC are limited, immunotherapy has attracted increasing attention. However, depending on the different patterns of tumor-infiltrating immunocytes, individuals respond significantly differently after immunotherapy.

A number of studies have found that differential expression of IRGs affects tumor prognosis and response to immunotherapy, probably because these genes cause different levels of infiltration of various immune cell subtypes in tumors^14–17^. The interaction of the tumor with its microenvironment is crucial in the development and progression of the tumor. A comprehensive analysis of the expression of immune-related genes and the functional roles of different subsets of tumor-infiltrating cells in the tumor immune microenvironment could improve our knowledge of immunology and define subgroups of patients who are more likely to respond to immunotherapy. Therefore, in this study, we identified the survival-associated IRGs that were significantly related with the development and progression of HNSCC. In the functional enrichment analysis, one significant pathway identified was related to cytokine-cytokine receptor interactions in HNSCC, and the main enriched GO terms were immunocyte migration and activity of the receptor and ligand regardless of HPV status. The immune cell infiltration was mainly mediated by chemokine/receptor expression networks and cancer genetic alterations in tumor tissue via a systematic analysis of multiple cancers^18^. As expected, biological functions of the identified genes were significantly associated with inflammation progression. The results suggested that these differentially expressed genes were mostly enriched in inflammation-related terms and pathways.

Based on this result, we selected 10 genes (PLAU, SFTPA2, CCL26, SEMA3G, DKK1, GAST, GNRH1, PDGFA, ZAP70, and STC1) and 11 genes (SEMA3G, GNRH1, TNFRSF4, ZAP70, PLAU, SH2D1A, CCL26, DKK1, GAST, PDGFA and STC1) that were closely related to clinical prognosis to construct two prognostic prediction models to assess potential clinical outcomes for the total HNSCC patients and HPV- HNSCC patients. Notably, GNRH1 has been suggested as a marker of the metastatic spread of gynecological cancer^19^. PDGFA, a member of the platelet-derived growth factor (PDGF) family, may play a crucial role in the composition of the immune microenvironment^20^. PLAU, one of the major proteolytic enzymes involved in the degradation of extracellular matrix, has been demonstrated to play a critical role in tissue remodeling and migration in the developmental of cancer and in tumorigenesis^21^. Some studies indicated that PLAU was a marker to predict OS in HNSCC patients^22^. Stanniocalcin-1 (STC1) is a secreted glycoprotein implicated in several pathologies, including inflammation and cancer. Several studies have shown that STC1 is associated with cancer development^23,24^. It was verified that STC1 could accelerate tumor growth and reduce disease-free survival in mice. SEMA3G is a member of the class 3 semaphorin family originally characterized in axonal guidance^25^. Semaphorins have been shown to play multiple roles in normal and pathologic angiogenesis by acting on their receptors, plexins and neuropilins^26^. Methylation of the SFTPA2 promoter represents a potential biomarker for lung cancer diagnosis. The SFTPA2 DNA methylation profile was used as a potential tool to monitor disease progression and immunity^27^. The immune-related gene ZAP70 was associated with an increased risk of developing virally mediated head and neck squamous cell carcinoma. Mutations in many of these genes have previously been implicated in cancer risk, viral host-response, or epithelial immunity^28^. Gastrin is a growth factor of the gastrointestinal mucosa, and its role in gastrointestinal tumorigenesis is well studied. High levels of gastrin have been correlated with the poor prognosis of lung cancer patients. Gastrin-releasing peptide (GRP) signaling appears to mediate the autocrine growth of human squamous cell carcinoma of the head and neck^29,30^. Dickkopf-1 (DKK-1) is a secreted protein, and the expression and DKK-1 is different in various cancers. The methylation of DKK1 may be considered a prognostic marker in oral cancer^31^. DKK1 knockdown increased cellular migration and invasiveness in oral cancer cells^32^. High expression of DKK-1 was associated with poor prognosis, and this suggests that DKK-1 may be a useful molecular marker in breast cancer^33^. OChemokine ligand 26 (CCL26) levels were elevated in and positively correlated with stage III and IV colorectal cancer (CRC) tissues and were associated with a poor prognosis in CRC patients^34^. TNFRSF4 (also known as OX40 or CD134) is a member of the tumor necrosis factor receptor superfamily expressed on activated T cells^35^. TNFRSF4 has also been shown to promote T cell survival, proliferation, and memory, enhance cytokine secretion, thus further enhance antitumor immunity^36^. Therefore, the quality of T cells is as important as T cell number in response to HMSCC immunotherapy. SH2 domains are commonly found in adapter proteins that aid in the signal transduction of receptor tyrosine kinase pathways^37^. SH2D1A is also a SH2 domain-containing protein, which mutations caused the X-linked lymphoproliferative syndrome (XLP) and was associated with B-cell lymphomas^38^. These findings indicate the potential clinical application of IRGs as biomarkers in prognosis prediction and the promise of HNSCC immunotherapy. Our study will also provide a bioinformatics evidence for the prognosis prediction of HPV- HNSCC patients.

In univariable and multivariable Cox regression analyses, interestingly, we found that the expression of prognostic immune-related genes (CD79A, NR3C2, and PDGFRB) was significantly correlated with the sex of HNSCC patients. The results indicated that the immune microenvironment and the levels of infiltration by immune cells may be different in male and female HNSCC patients. Thus, male and female patients respond differently to immunotherapy. Recent findings reported that immune checkpoint inhibitors were twice as effective as standard cancer therapies in the treatment of men with advanced solid tumors compared to their female counterparts^39,40^. Immune checkpoint inhibitors can improve overall survival for patients of both sexes with some types of advanced cancers, but men have a greater treatment effect from these drugs versus control treatments than do women^41^. Sex differences in congenital and adaptive immune responses are known, and women generally have a stronger immune response than men. Therefore, different treatment strategies are adopted for patients of different sexes. The immune microenvironment should be improved in male cancer patients, while tumor antigenicity should be enhanced in female cancer patients^42,43^. However, Wallis *et al*. demonstrated no statistically significant association of patient sex with the efficacy of immunotherapy in the treatment of advanced cancers using overall survival as the outcome via meta-analysis^44^.

HNSCC represents a group of tumors occurring at various sites, including the oral mucosa and the palatine tonsils. Adding to this diversity is the recent observation that a proportion of these cancers, notably tonsillar carcinomas, are associated with human papillomavirus (HPV) infection^41^. HPV has been implicated in the etiology of a subset of HNSCCs, which often arise in younger patients without a history of alcohol or tobacco use^42^. Approximately 30% of HNSCC tumors are HPV^+^, and despite late-stage presentation, they often have a better prognosis and response to therapy^45^. A high level of CD8^+^ T-cell infiltration might be an important factor contributing to the improved survival of HPV^+^ HNSCC patients^46^. In current study, we also identified 13 differentially expressed IRGs that were significantly associated with overall survival (OS) of the HPV + HNSCC patients (Tables S2, 3; Fig. S1). However, the number of HPV + HNSCC samples are not enough for prognosis and immune cell infiltration analyses.

Tumor-infiltrating lymphocytes (TILs) are generally thought to represent a host immune response directed against antigens expressed on tumor cells, and a high density of TILs has been identified as a favorable marker in HNSCC^47^. Studies of HNSCC and other cancers have suggested a beneficial effect of B cells on outcome^48–50^. However, little is known about the role of TILs in the development of head and neck squamous cell carcinoma. No research has reported the relationship between the expression of IRGs and TIL status. Thus, knowledge of immune infiltration is important for an in-depth understanding of tumor and immune interactions. We investigated the relationship between prognosis-related immune gene expression and immune cell infiltration to determine the status of the HNSCC immune microenvironment and found that B cell and CD4 T cell infiltration levels were significantly negatively correlated with the expression of prognosis-related immune genes in total HNSCC patients. However, B cell, CD4 T cell, CD8 T cell, dendritic cell and macrophage infiltration levels were significantly negatively correlated with the risk score of the HPV- patients. IRGs were significantly associated with B cell infiltration in lung adenocarcinoma (LUAD), and these genes are involved in tumor immunity and may play an important role in the prognosis of patients^51^. Lin *et al*. demonstrated that an IRG-based risk score was significantly negatively correlated with the infiltration of CD4 + T cells, B cells and macrophages^52^. Similarly, our results confirmed a low level of infiltrating B cells and CD4 + T cells in high-risk patients who had a poor prognosis regardless of HPV status.

This study comprehensively analyzed IRGs and the TME based on data from TCGA. Differentially expressed IRGs were proposed to determine the abundance of infiltrating immune cells and assess potential clinical outcomes. We will aim to validate the molecular mechanism and further in-depth analysis the impact of different HPV status on the prognosis of HNSCC patients in future studies, particularly HPV + HNSCC patients.

Inevitably, there were several limitations in our study. The current results lack prognostic analysis of HPV + HNSCC patients due to small number of HPV + samples, and the lack of validation in a prospective clinical trial is also a limitation of the study. Moreover, the mechanism by which the expression of the prognosis-related IRGs affects the outcome of HNSCC patients remains unclear.

In conclusion, we arrived at a more comprehensive understanding of the TME and created a list of prognostic immune‐related genes with the potential to become prognostic biomarkers.

## Materials and Methods

### Clinical samples and immune gene data

All data in this study were obtained from a public database. The transcriptome profiling data of HNSCC samples were downloaded from the TCGA data portal (https://portal.gdc.cancer.gov/), which contained data from 501 primary HNSCC and 44 nontumor samples. The clinical information of 527 HNSCC patients was extracted and downloaded for further analysis. We also identified immune-related genes via the Immunology Database and Analysis Portal (ImmPort) (https://www.immport.org/) (Table S1). ImmPort is an archival repository and dissemination platform for clinical and molecular datasets. ImmPort is an important source of raw data and protocols from clinical trials, mechanistic studies, and novel methods for cellular and molecular measurements^53^. In addition, ImmPort is also a database that updates real-time data accurately and provides a list of IRGs that are actively involved in tumor immunological processes for cancer research.

### Differential gene analysis

We performed differential gene analysis on all transcriptome profiling data using the R software limma package (http://www.bioconductor.org/packages/release/bioc/html/limma.html), with a false discovery rate (FDR) < 0.05 and log2 |fold change | > 1 as the cutoff values. Heatmaps were generated using the pheatmap package in R. Then, we extracted differentially expressed IRGs from the intersection of immune genes and all differentially expressed genes. To explore the potential molecular mechanisms of differentially expressed IRGs, functional enrichment analysis was performed via GO and KEGG pathway analyses^54–56^ using the R software clusterprofiler package^57^.

### Survival-related IRGs and survival analysis

We used Perl software to analyze the clinical characteristics and follow-up data downloaded from TCGA and chose overall survival (OS) as the primary endpoint. Survival-related genes were selected by univariable COX regression analysis (FDR < 0.05). We made proportional hazards assumptions based on Schoenfeld residuals (phtest) for the COX regression model. The significance value for the overall test of proportional hazards is less than 0.05 (P < 0.05). Hazards ratio (HR) is the ratio of tumor samples and normal samples IRGs expression. We defined the high-risk IRGs (HR > 1) and low-risk IRGs (HR < 1) with HR = 1 as a cutoff. The Kaplan-Meier survival curve was drawn using the R software survival package according to a significance filter of P < 0.05. Functional enrichment analysis was also performed on survival-related IRGs that were significantly associated with OS.

### Construction of the immune gene-related prognostic model

The expression data of these survival-related genes and their coefficients were used to develop a gene-based prognosis prediction model, and Akaike information criterion (AIC) was used to build the model in order to avoid the data of overfitting, with the minimum AIC value representing the target model^58^. The formula used is as follows: ${\rm{risk}}\,{\rm{score}}={\sum }_{{\rm{i}}=1}^{{\rm{n}}}{\rm{coefgenei}}\times {\rm{expgenei}}$^59^. Multivariable Cox regression analysis was used to illustrate the correlation of risk scores with patient overall survival (OS) and to identify potential prognostic genes. Patients were divided into high- and low-risk groups based on the median risk score value.

### Correlation analysis of the clinical data

Relationships were analyzed between single genes and clinical factors via the R software beeswarm package. In addition, we also used clinical characteristics for univariable and multivariable Cox regression analyses with the R software survival package. The risk scores were divided into high- and low-risk groups based on median risk score, and ages were divided into ≤65 group and >65 group. The grades were divided into G1–2 and G3, stages were divided into I-II and III-IV groups, stage T were divided into T1–2 and T3–4 groups, stage N were divided into N0 and N1–3 groups and stage M were divided into M0 and M1 groups. We selected age (>65), sex (female), grade (G1–2), stage (I-II), T (1–2), M (M0) and N (N0) as the reference for each group. Risk score P-values of less than 0.001 were considered statistically significant.

### Clinical relevance of tumor immune infiltration

We downloaded the immune infiltration levels of HNSCC patients using the Tumor IMmune Estimation Resource (TIMER) (http://cistrome.org/TIMER/), which is a web resource for the systematic evaluation of the clinical impact of different immune cells in diverse cancer types. We used this resource to investigate possible associations between the abundance of six subtypes of tumor-infiltrating immune cells (B cells, CD4 T cells, CD8 T cells, macrophages, neutrophils, and dendritic cells) and patient prognosis. Based on the immune gene-related prognostic model, we performed a correlation analysis combining the risk score and immune cell infiltration level of each sample via R software.

### Transcription factor (TF) regulatory network

To explore the interactions between high-risk survival-associated IRGs and transcription factors (TFs), we obtained data on 318 TFs from the Cistrome online database (https://cistrome.org/). Cistrome is a comprehensive database for cancer transcription factor (TF) targets and provides regulatory links between TFs and transcriptomes. A protein–protein interaction (PPI) network was constructed based on data gleaned and displays many direct and indirect interactions with genes. The resulting PPI network was analyzed with Cytoscape software version 3.7.2. We extracted differential TFs to construct the regulatory network of the high-risk survival-associated IRGs (HR > 1) and potential TFs.

### Statistical analysis

Differential analysis of immune genes, function enrichment analysis, COX regression analysis and survival analysis were performed using R software (version 3.6.1) and R packages. The Kaplan–Meier curve was created with the R survival and survminer packages. Based on the survival ROC R software package, the AUC of the survival ROC curve was calculated to verify the accuracy of the prognosis prediction model. Differences between clinical characteristics and prognosis-related IRGs were tested using an independent Student’s t test. P-values of less than 0.05 were considered statistically significant.

## Supplementary information

Supplementary information.

Supplementary information2.

Supplementary information3.

Supplementary information4.

Supplementary information5.

Supplementary information6.

Supplementary information7.

Supplementary information8.

Supplementary information9.

Supplementary information10.
