# Supplementary material for: Comprehensive immunogenomic landscape analysis of prognosis-related genes in head and neck cancer
Source: Sci Rep. 2020 Apr 14;10:6395. doi: 10.1038/s41598-020-63148-8 (PMC7156482; doi:10.1038/s41598-020-63148-8)
Supplement: Supplementary file 6 — Supplementary information6. [file 41598_2020_63148_MOESM6_ESM.pdf]

Comprehensive immunogenomic landscape analysis of prognosis-related genes in head and neck cancer

Lei Li[1*], Xiao-Li Wang[2*], Qian Lei[1], Chuan-Zheng Sun[1], Yan Xi[1], Ran Chen[1], Yong-Wen He[3]

[1]Department of Head and Neck Surgery Section II, the Third Affiliated Hospital of Kunming Medical University, 519 Kunzhou Road, Kunming, China

[2]Radiation Therapy Center, the Third Affiliated Hospital of Kunming Medical University, 519 Kunzhou Road, Kunming, China

[3]Department of Dental Research, The Affiliated Stomatological Hospital of Kunming Medical University, Yunnan, China

Correspondence

Y. He, Department of Dental Research, The Affiliated Stomatological Hospital of Kunming Medical University, Block C, No. 1088 Haiyuan Middle Road, High and New Technology Zone, Kunming 650000, Yunnan, China

Fax: +86 0871 5330099

Tel: +86 1366 8796269

E-mail: k92oxu@163.com or heyongwen2@sina.com

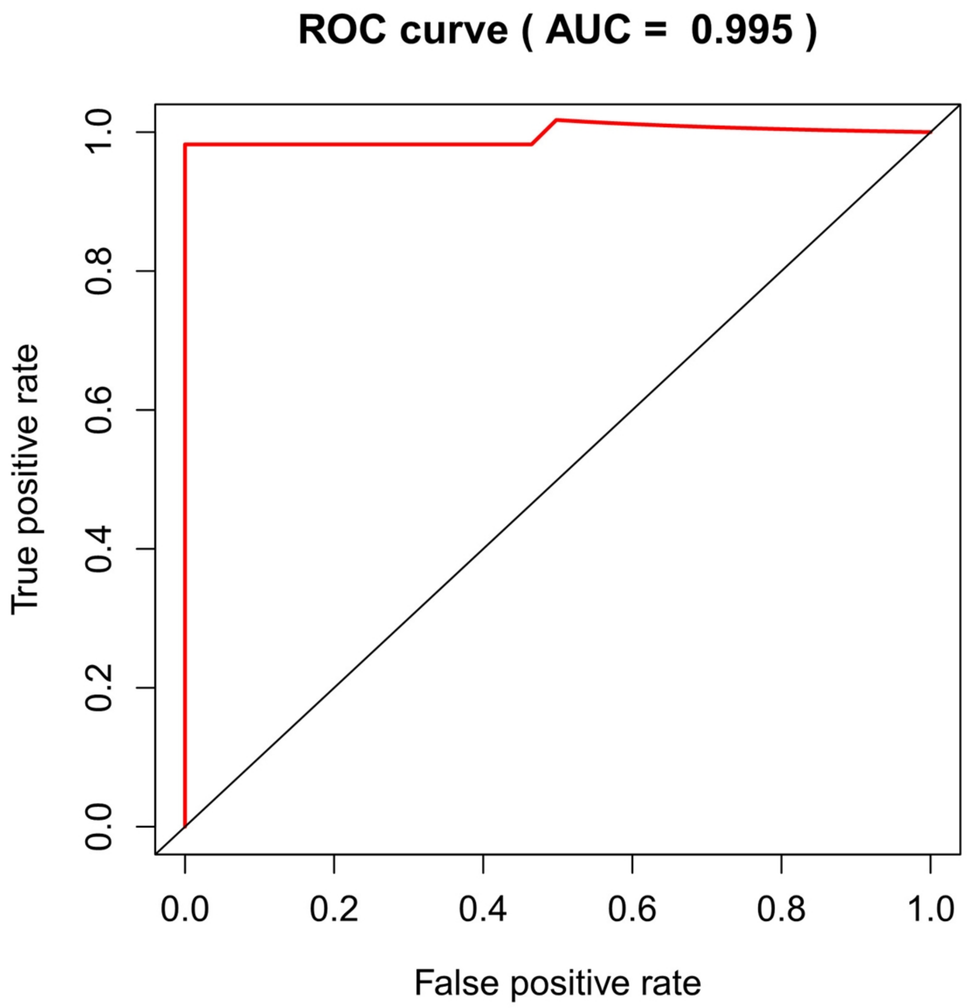
ROC curve (AUC = 0.995)
True positive rate
0.0
0.2
0.4
0.6
0.8
1.0
False positive rate
0.0
0.2
0.4
0.6
0.8
1.0
