# Supplementary material for: Comprehensive immunogenomic landscape analysis of prognosis-related genes in head and neck cancer
Source: Sci Rep. 2020 Apr 14;10:6395. doi: 10.1038/s41598-020-63148-8 (PMC7156482; doi:10.1038/s41598-020-63148-8)
Supplement: Supplementary file 7 — Supplementary information7. [file 41598_2020_63148_MOESM7_ESM.pdf]

Comprehensive immunogenomic landscape analysis of prognosis-related genes in head and neck cancer

Lei Li[1*], Xiao-Li Wang[2*], Qian Lei[1], Chuan-Zheng Sun[1], Yan Xi[1], Ran Chen[1], Yong-Wen He[3]

[1]Department of Head and Neck Surgery Section II, the Third Affiliated Hospital of Kunming Medical University, 519 Kunzhou Road, Kunming, China

[2]Radiation Therapy Center, the Third Affiliated Hospital of Kunming Medical University, 519 Kunzhou Road, Kunming, China

[3]Department of Dental Research, The Affiliated Stomatological Hospital of Kunming Medical University, Yunnan, China

Correspondence

Y. He, Department of Dental Research, The Affiliated Stomatological Hospital of Kunming Medical University, Block C, No. 1088 Haiyuan Middle Road, High and New Technology Zone, Kunming 650000, Yunnan, China

Fax: +86 0871 5330099

Tel: +86 1366 8796269

E-mail: k92oxu@163.com or heyongwen2@sina.com

Figure S1. (A) Forest plot of hazard ratios show the prognosis-related immune genes in HPV+ HNSCC patients. Red dots represent high risk genes (HR>1), green dots represent low risk genes (HR<1). (B) Association between risk score and the distribution of high (red) and low (green) risk groups. (C) Survival status of the HPV+ HNSCC patients in different groups; red and green dots represent deceased and surviving patients, respectively. (D) Heat map representing the expression level of the prognostic genes based on the high-risk group and the low-risk group. (E) The overall survival rates of different groups of HPV+ HNSCC patients. (F) Verification of the accuracy of the prognostic model via analysis of the area under curve (AUC) of the receiver operating characteristic (ROC) curve.

Table S1. The list of immune-related genes

Table S2. Identification of the survival-related IRGs (The logFC means log2 |fold change|, log F/C >1 indicated that the expression of gene was up-regulated in HPV+ HNSCC patients, while log F/C <1 indicated that expression of gene was down-regulated in HPV+ HNSCC patients). The FDR means the false discovery rate. Tumor mean represented the average gene expression in tumor samples and normal mean represented the average gene expression in normal samples.

Table S3. Relationships between the expression of immune-related genes and overall survival in HPV+ HNSCC patients. Immune-related genes were divided into high risk and low risk the prognosis-related genes via HR value (HR>1 indicated high risk and HR<1 indicated low risk).
