# Supplementary material for: Comprehensive immunogenomic landscape analysis of prognosis-related genes in head and neck cancer
Source: Sci Rep. 2020 Apr 14;10:6395. doi: 10.1038/s41598-020-63148-8 (PMC7156482; doi:10.1038/s41598-020-63148-8)
Supplement: Supplementary file 10 — Supplementary information10. [file 41598_2020_63148_MOESM10_ESM.pdf]

Comprehensive immunogenomic landscape analysis of prognosis-related genes in head and neck cancer

Lei Li[1*], Xiao-Li Wang[2*], Qian Lei[1], Chuan-Zheng Sun[1], Yan Xi[1], Ran Chen[1], Yong-Wen He[3]

[1]Department of Head and Neck Surgery Section II, the Third Affiliated Hospital of Kunming Medical University, 519 Kunzhou Road, Kunming, China

[2]Radiation Therapy Center, the Third Affiliated Hospital of Kunming Medical University, 519 Kunzhou Road, Kunming, China

[3]Department of Dental Research, The Affiliated Stomatological Hospital of Kunming Medical University, Yunnan, China

Correspondence

Y. He, Department of Dental Research, The Affiliated Stomatological Hospital of Kunming Medical University, Block C, No. 1088 Haiyuan Middle Road, High and New Technology Zone, Kunming 650000, Yunnan, China

Fax: +86 0871 5330099

Tel: +86 1366 8796269

E-mail: k92oxu@163.com or heyongwen2@sina.com

Gene	HR	HR95%CI	HR95%CI	P value
PDIA2	1.9333939	1.1345263	3.2947778	0.0153492
CXCL2	1.0683744	1.0094507	1.1307375	0.0223167
IL1B	1.0842039	1.0034858	1.1714147	0.0405484
SFTPA2	4.8136268	1.4775263	15.682294	0.0091135
BIRC5	1.0875321	1.0190678	1.1605961	0.0114287
IGLV3-21	1.0013302	1.0000071	1.002655	0.0487794
CD70	1.0344991	1.004435	1.0654632	0.0241933
CSPG5	2.0452826	1.0498753	3.9844547	0.0354646
IL12A	1.8495177	1.0927576	3.1303519	0.0220009
INHA	43.673222	1.0711101	1780.723	0.0459041
IL22RA2	1.3660737	1.0217743	1.8263892	0.0352613
SSTR2	1.1725153	1.0121521	1.3582862	0.0339274
TEC	19.620388	1.1897086	323.57473	0.0373942
